# A diverse virome in kidney transplant patients contains multiple viral subtypes with distinct polymorphisms

**DOI:** 10.1038/srep33327

**Published:** 2016-09-16

**Authors:** Asha Rani, Ravi Ranjan, Halvor S. McGee, Ahmed Metwally, Zahraa Hajjiri, Daniel C. Brennan, Patricia W. Finn, David L. Perkins

**Affiliations:** 1 Department of Medicine, University of Illinois, Chicago, IL 60612, USA; 2 Department of Bioengineering, University of Illinois, Chicago, IL 60612, USA; 3 Division of Renal Diseases, Washington University School of Medicine, St. Louis, MO 63110, USA; 4 Department of Surgery, University of Illinois, Chicago, IL 60612, USA

## Abstract

Recent studies have established that the human urine contains a complex microbiome, including a virome about which little is known. Following immunosuppression in kidney transplant patients, BK polyomavirus (BKV) has been shown to induce nephropathy (BKVN), decreasing graft survival. In this study we investigated the urine virome profile of BKV+ and BKV− kidney transplant recipients. Virus-like particles were stained to confirm the presence of VLP in the urine samples. Metagenomic DNA was purified, and the virome profile was analyzed using metagenomic shotgun sequencing. While the BK virus was predominant in the BKV+ group, it was also found in the BKV− group patients. Additional viruses were also detected in all patients, notably including JC virus (JCV) and Torque teno virus (TTV) and interestingly, we detected multiple subtypes of the BKV, JCV and TTV. Analysis of the BKV subtypes showed that nucleotide polymorphisms were detected in the VP1, VP2 and Large T Antigen proteins, suggesting potential functional effects for enhanced pathogenicity. Our results demonstrate a complex urinary virome in kidney transplant patients with multiple viruses with several distinct subtypes warranting further analysis of virus subtypes in immunosuppressed hosts.

Kidney transplantation is the preferred treatment modality for most patients with end stage renal disease (ESRD) and offers an enhanced lifestyle and longer survival for many patient groups^1^. With current kidney transplantation protocols, the rate of graft survival in many centers is more than 80% at five years from a deceased donor and even higher for living donors^2^. However, transplantation requires life-long immunosuppression that leads to side effects including an increased incidence of infections. Viruses are one major cause of such infections. Immunosuppressed patients may develop *de novo* viral infections or reactivation of latent viral infections. Prominent examples in the latter category include BK virus (BKV) and JC virus (JCV), both members of the *Polyomaviridae* family. Primary infection with BKV and JCV occurs in healthy individuals, often during childhood, and is detected by sero-positivity of anti-viral antibodies. Following infection, these viruses commonly becomes latent; however, they persist in the urinary tract and can reactivate in the context of immunosuppression following transplantation^3^,^4^. Previous studies have established that polyoma viruses infect the kidney and urinary tract and can lead to impaired renal function or even graft failure. Polyomavirus associated nephropathy develops in approximately 10% of the kidney transplant patients, resulting in kidney dysfunction with graft failure in 15–50% of affected grafts^5^,^6^,^7^,^8^. In kidney transplantation, it is unclear whether BKV infection is transferred from donor to recipient or reactivated by immunosuppression in the recipient. Notably, a recent study has suggested that the BKV originates from the donor kidney^9^.

The majority of BKV and JCV infections are diagnosed by quantitative PCR in the urine and blood or by use of decoy cells (cytology)^6^,^10^,^11^. While these established methods are routinely employed for clinical diagnosis, they are limited in identifying multiple virus subtypes and cohabiting latent viruses which may contribute to graft dysfunction or failure. Virus detection methods based on mass spectrophotometry have also been reported to differentiate and identify the BKV subtypes from urine samples^12^. With recent advancements in high-throughput shotgun metagenome sequencing it is now possible to identify nearly complete virus genomes, including genetic polymorphisms and virus subtypes, with high accuracy directly from clinical samples.

While the virome has been less studied than its bacterial counterpart in human microbiome analyses^13^, it is an important component and contributes significantly to human disease^14^,^15^. For example, the role of the gut virome is now appreciated in maintaining homeostasis in disease^16^,^17^. The virus metagenomes identified in this study by shotgun metagenomics will aid in further identification and characterization of different virus subtypes and genetic polymorphisms. There are four main BKV subtypes (I–IV) based on genome sequences, with subtype I further divided into four sub-groups (Ia, Ib-1, Ib-2 and Ic)^18^. JCV has 14 described subtypes associated with various demographic variables^19^. The specific nucleotide substitutions which alter amino acid residues in core proteins may play a critical role in the pathogenicity, antigenicity, receptor specificity and viability of the viruses^20^,^21^,^22^. In this study, we investigated the urinary virome to determine the specificity and abundance of viruses present in the urine following kidney transplantation.

## Results

### Patient demographics and viral metagenomics

**Figure 1:**
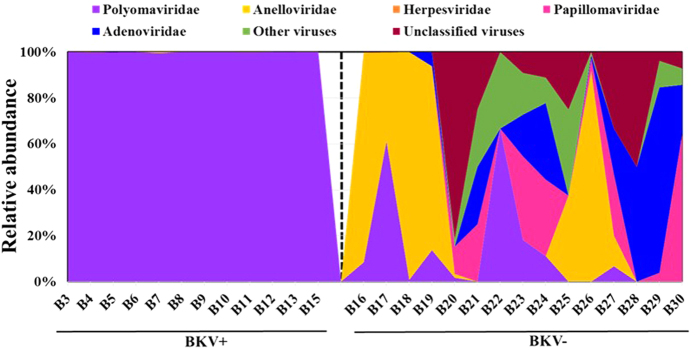
Virus diversity in the urine of transplant patients. Stacked area chart representation of virus at family taxonomic level identified in BKV+ and BKV− group. *Polyomaviridae* was the most dominant family of viruses identified in the BKV+ group, and *Anelloviridae* was dominant in the BKV− group. The BKV− group was highly variable and a patient specific virus profile was observed.

We compared kidney transplant patients with a clinical diagnosis of BK viremia with patients that were negative for viremia based on clinical diagnosis by serum PCR titers. The demographics and clinical diagnoses of the 22 patients are representative of the clinical characteristics of the general transplant population (Table 1). To confirm the detection of VLP in the urine, we stained the urine samples with SyBr Gold and performed epifluorescence microscopy. We detected VLP [dim pinpoints, denoted by blue arrows], and also the microbial cells [larger and brighter, denoted by red arrows] (Supplementary Figure S1a). Similar observation about detecting VLP and microbial cells by epifluorescence microscopy in samples have been reported earlier^23^. Next, we purified metagenomic DNA, constructed libraries and performed metagenomic shotgun sequencing on Illumina HiSeq 2000. The reads were filtered to remove human sequences, and as expected, we detected significantly increased numbers of virus reads in the BKV+ samples (6,020,000 ± 1,103,000) compared to BKV− samples (132,500 ± 81,260) (*p* < 0.001, Supplementary Figure S1b). We identified comparable numbers of non-human and non-virus sequence reads in the BKV+ (3,479,000 ± 1,282,000) and BKV− (2,429,000 ± 1,234,000) samples (Supplementary Figure S1c). Next, the virus sequence reads and the non-virus reads from BKV+ and BKV− samples were filtered in silico. The reads in the BKV+ samples included large numbers of virus sequences, but also contained bacterial, eukaryotic and unclassified sequences (Supplementary Figure S1d). Among the patients negative for viremia, 4 had clinically diagnosed BK viruria, which is consistent with studies showing that viruria often precedes viremia^24^. There was no significant correlation between clinical parameters such as age, BMI (Body Mass Index) or weight, and the status of viremia or viruria (Supplementary Figure S2). We calculated the relative abundance of the urinary virome in the kidney transplant patients (Fig. 1a). In addition to BKV, which was the predominant virus in the BKV+ patients, we also detected *Anelloviridae, Adenoviridae, Herpesviridae, Papillomaviridae* and small numbers of unclassified viruses (Supplementary Table S1). A statistically significant difference was observed in total BKV counts between the BKV+ group and the BKV− group (Supplementary Figure S1e), as expected, but no significant difference was observed for JCV and TTV, although a trend towards higher overall virus counts in the BKV− group was noted (Supplementary Figure S1f and g). In the BKV− patients, the *Anelloviridae* subtypes, which had the second highest relative prevalence following BKV, were detected in 53% of patients. In addition, Adenoviruses were detected in 37%, Papillomaviruses in 33% and *Herpesviridae* in 15% of the patients.

**Figure 2:**
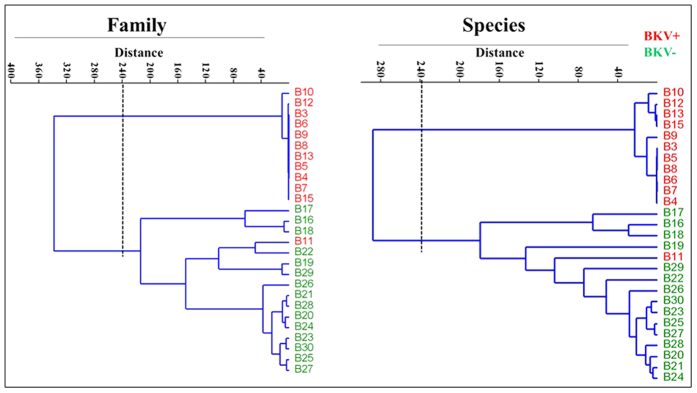
Cluster analysis of the urine virome. The cluster analysis is based on hierarchical cluster using the Ward’s method with the Pearson’s correlation distance metric at taxonomic rank of the virus family and species identified in the samples. Each line in the cluster corresponds to a sample name and is color coded to show BKV+ (red) and BKV− (green) group.

**Figure 3:**
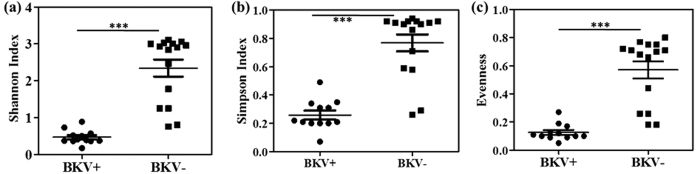
Virus diversity index measures in the BKV+ and BKV− groups. (**a**) Shannon diversity, (**b**) Simpson diversity and (**c**) Evenness indices. A significant difference was observed in all the diversity measures among the BKV+ and BKV− groups. The BKV− group represents the highest virus diversity and evenness of virus species, and the indices were highly variable among the group compared to BKV+ group. The BKV+ group represented less virus diversity and low evenness among the samples due to a high BKV count among the group. The difference was computed using Welch *t*-test, ^***^p < 0.001.

**Figure 4:**
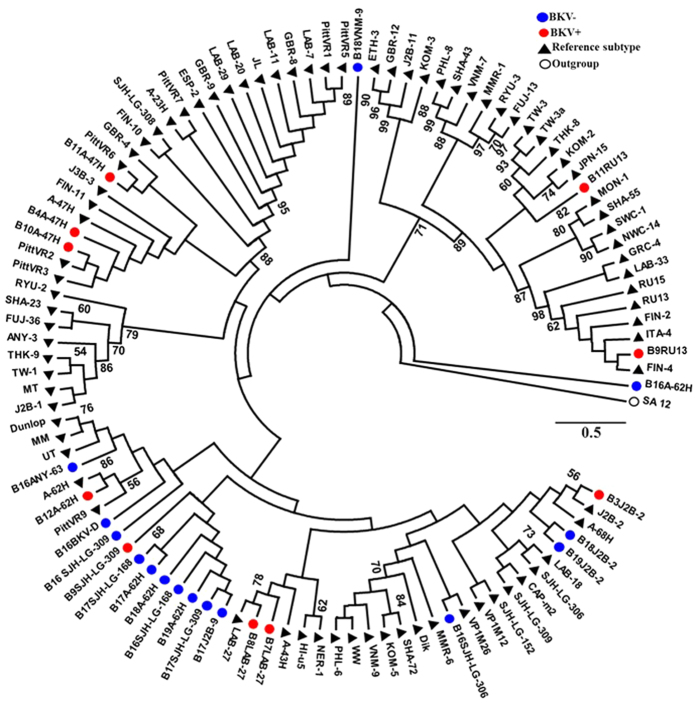
Phylogenetic analysis of BK virus. Phylogenetic tree based on the reference genomes of 81 BKV strains. The genomes from the BKV+ (red) and BKV− (blue) subjects are intercalated into the circular dendograms. The evolutionary history of the virus strains was inferred by the maximum likelihood method based on the Tamura-Nei model. The bootstrap consensus trees were inferred from 100 replicates that represent the evolutionary history of the virus taxa. Branches corresponding to partitions reproduced in less than 50% bootstrap replicates are collapsed. SA12 (simian agent 12, a baboon polyoma virus) was used as an out group. The tree is drawn to scale, with branch lengths measured in the number of substitutions per site. Samples analyzed in the study are labelled as - sample name/isolate name.

**Figure 5:**
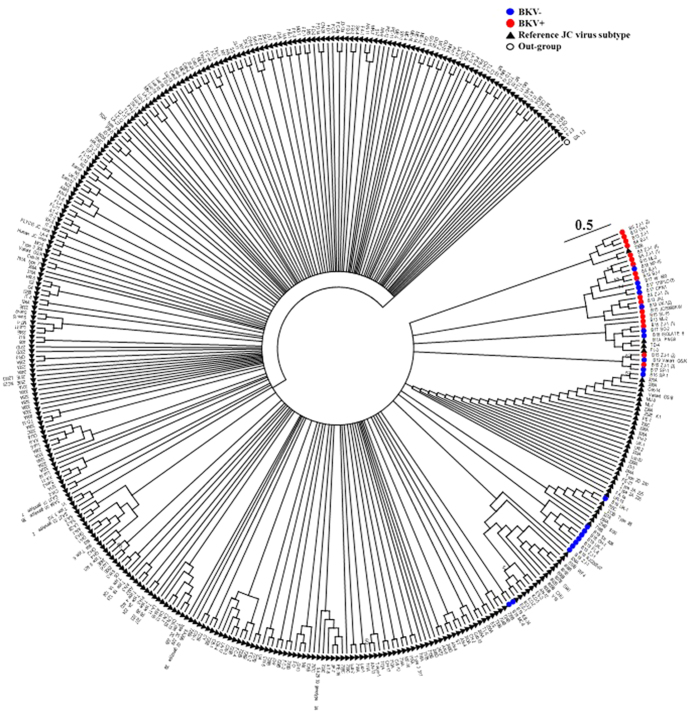
Phylogenetic analysis JC virus. Phylogenetic tree based on the reference genomes of 333 JCV strains. The genomes from the BKV+ (red) and BKV− (blue) subjects are intercalated into the circular dendograms. The evolutionary history of the virus strains was inferred by the maximum likelihood method based on the Tamura-Nei model. The bootstrap consensus trees were inferred from 100 replicates that represent the evolutionary history of the virus taxa. Branches corresponding to partitions reproduced in less than 50% bootstrap replicates are collapsed. SA12 (simian agent 12, a baboon polyoma virus) was used as an out group. The tree is drawn to scale, with branch lengths measured in the number of substitutions per site. Samples analyzed in the study are labelled as - sample name/isolate name.

**Figure 6:**
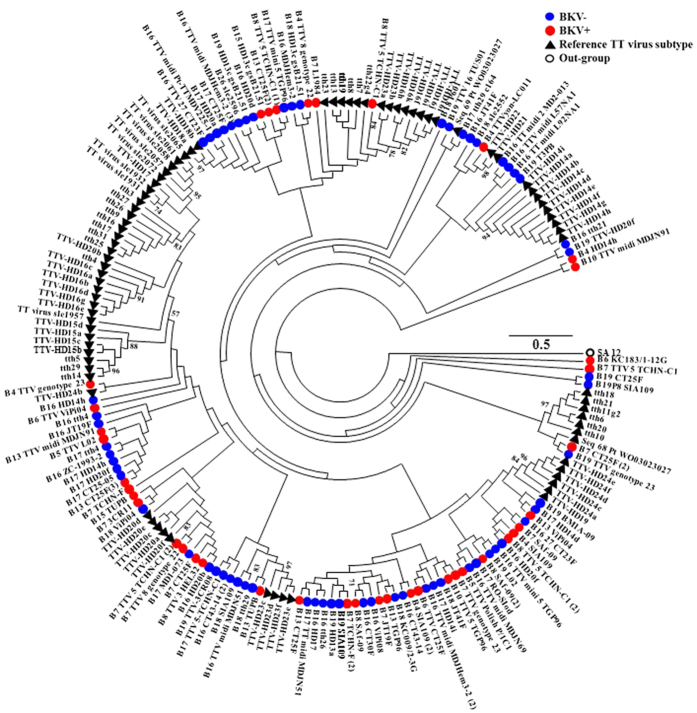
Phylogenetic analysis TT virus. Phylogenetic tree based on the reference genomes of 93 TTV strains. The genomes from the BKV+ (red) and BKV− (blue) subjects are intercalated into the circular dendograms. The evolutionary history of the virus strains was inferred by the maximum likelihood method based on the Tamura-Nei model. The bootstrap consensus trees were inferred from 100 replicates that represent the evolutionary history of the virus taxa. Branches corresponding to partitions reproduced in less than 50% bootstrap replicates are collapsed. SA12 (simian agent 12, a baboon polyoma virus) was used as an out group. The tree is drawn to scale, with branch lengths measured in the number of substitutions per site. Samples analyzed in the study are labelled as - sample name/isolate name.

**Figure 7:**
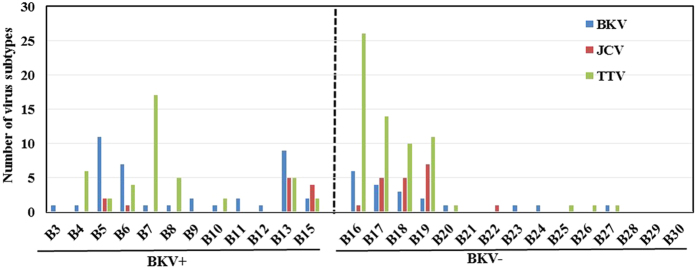
Identification of virus subtypes. The bar chart represents the number of BKV, JCV, and TTV subtypes identified in each patient, In the BKV+ group, at least one virus subtype was detected in each patient, and multiple subtypes of other virus were detected in some patients in BKV+ group. In the BKV− group, four samples were detected that contained subtypes of all 3 viruses including BK, JC and TT virus.

The BK virus family contains subtypes specific for animal species ranging from mice to chimpanzees. In a hierarchical clustering analysis of 26 species-specific BKV subtypes (as described previously^25^), our sequences, e.g. the B19/A-62H (1a) sequences, clustered adjacent to the human BKV Dunlop (1a) reference subtype confirming the species specificity of the isolates (Supplementary Figure S3). A clustering analysis at either the family or species level separated the BKV+ and BKV− patients with the exception of patient B11, which had only 31.9% abundance of BKV and clustered with the BKV− subjects (Fig. 2). We next compared the diversity of the viruses in the BKV+ and BKV− groups based on the Shannon Index, Simpson Index and Evenness algorithms (Fig. 3a–c). All 3 indices show significantly decreased diversity in the BKV+ samples (*p* < 0.001). We found multiple virus subtypes in several individual patients. In an analysis of BKV subtypes, we mapped the 25 subtypes identified in our sequence data onto the 81 known BKV reference genomes^26^ in a circular dendrogram, calculating the sequence similarity using the Tamura-Nei model calculated by the Maximum Likelihood method (Fig. 4). The most common subtypes of BKV in our patients were Ib1 and Ia, with lower numbers of subtypes Ib2, IV and Ic. In a similar analysis of JC virus, we mapped the clinical isolates to 333 previously characterized JC subtypes^19^ and showed that our clinical isolates mapped to a small subset of the virus subtypes (Fig. 5). We similarly mapped multiple TTV subtypes in our samples to the known TTV reference genomes^27^ (Fig. 6). Interestingly, in some BKV positive samples we identified multiple virus subtypes with a maximum of 11 different subtypes of BKV in sample B5 (Fig. 7, Supplementary Tables S2a, S2b and S5). In fact, we detected a single BKV subtype in 6 samples in the BKV+ group and in 4 samples in BKV− group. The average number of BKV subtypes was 3.3 in the BKV positive group (Fig. 7, Supplementary Table S5). Multiple subtypes of the JCV were also identified based on genome sequences from individual samples, with a maximum of 7 subtypes identified in patient B19 (Supplementary Tables S3 and S5). Interestingly, TTV had the highest number (26) of subtypes present in a single patient (B16) (Supplementary Tables S4b and S5). Many patients have multiple subtypes of each virus (Fig. 7, and Supplementary Table S5). To confirm that the sequence reads were specific for BKV, JCV or TTV, we mapped the assembled contig sequences to a virus reference genome which confirmed that the sequences mapped to the breadth of the 5.5 kb BKV and JCV, and 3.5 kb of TTV reference genomes (Supplementary Figures S4a–c).

### Viral gene polymorphisms

**Figure 8:**
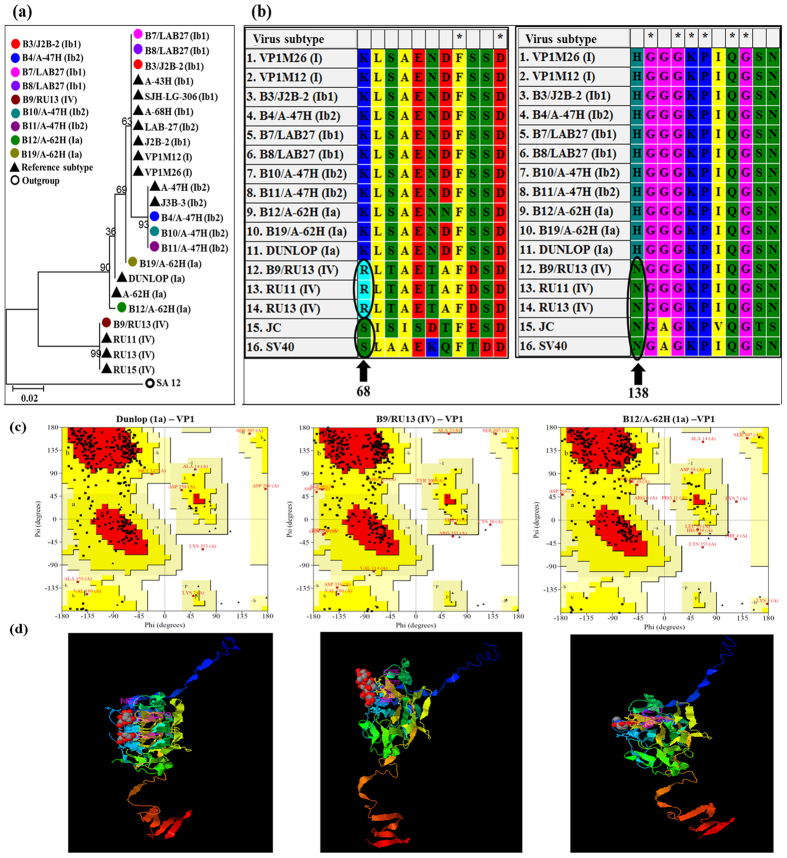
Analysis of the VP1 protein of BK virus. (**a**) Phylogenetic analysis of VP1. Phylogenetic tree based on the sequences of the VP1 region of the 14 reference BKV strains and 9 sequences described in this study. The evolutionary history was inferred by using the maximum likelihood method based on the Tamura-Nei model. The bootstrap consensus tree inferred from 100 replicates is taken to represent the evolutionary history of the taxa analyzed. The tree is drawn to scale, with branch lengths measured in the number of substitutions per site. SA12 (simian agent 12, a baboon polyoma virus) was used as an out group. (**b**) Multiple sequence alignment of VP1 protein reveals amino acid variations in VP1 protein. (**c**) The Ramachandran plot for the phi-psi torsion angles for residues in the VP1 protein of Dunlop (1a) (reference BKV subtype), B9/RU13 (IV) and B12/A-62H (1a) from BKV+ kidney transplant group. Glycine residues are separately identified by triangles. The coloring/shading on the plot represents the different regions described in Morris *et al*.^47^: the darkest areas (red) correspond to the “core” regions representing the most favorable combinations of phi-psi values. Over 90% of the residues in the “core” regions predicts a good quality structure model. The different regions on the Ramachandran plots are labelled as follows: A - Core alpha L - Core left-handed alpha a - Allowed alpha l - Allowed left-handed alpha ~a - Generous alpha ~l - Generous left-handed alpha B - Core beta p - Allowed epsilon b - Allowed beta ~p - Generous epsilon ~b - Generous beta. (**d**) Predicted 3D structure of the VP1 protein. For reference BKV isolate name is followed by subtype in parenthesis. Samples analyzed in the study are labelled as - sample name/isolate name (subtype).

Due to the presence of multiple virus subtypes, we detected polymorphisms in key proteins among the viruses in individual patient samples. This observation suggests that subtypes could differ in pathogenicity and clinical severity. To analyze polymorphisms in the BKV subtypes, we mapped the sequences of three key BKV genes and compared their resulting amino acid sequences. The VP1 gene mediates virus attachment to the cell-surface sialic acid on terminal alpha (2–8)-linked sialic acid-containing gangliosides GT1b and GD1b. Mapping VP1 sequences onto the phylogenetic tree of reference BK subtypes (Fig. 8a) gives a dendrogram showing 2 major plus several minor clusters of VP1 sequences which could modulate VP1 binding. Based on the crystal structure, residues 68 and 138 mediate VP1 interactions with cell surface receptors^20^. An alignment of the VP1 sequences identified polymorphisms in the VP1 protein at residues 68 and 138 containing non-conservative amino acid substitutions [Lys >Arg or Ser and His >Asp] (Fig. 8b). An almost complete BKV genome was sequenced from samples B9 and B12, with the shotgun metagenome sequencing approach, and BLAST analysis revealed that the BKV strains were similar to the RU13 (IV) and A-62H (1a) strains respectively. A complete multiple sequence alignment of the VP1 protein sequence among the BKV Dunlop (1a) strain and the BKV genomes of RU13 (IV) and A-62H (1a) revealed overall high similarity. Notably, there were several substitution in amino acids (Supplementary Figure S5). To assess the quality of VP1 predicted protein structures, Ramachandran (RM) plots were constructed. RM plots predict secondary structures from the dihedral angles of individual amino acids (phi-psi) and evaluate distributions of residues which are scored as degrees of favorability (most favored, additionally allowed, generously allowed, and not allowed regions). The RM plot statistics for the polymorphic VP1 proteins show that 70% of residues fall within favored regions, more than 25% fall within allowed regions and <5% are in external regions, indicating a high quality score for the protein structures of the polymorphic sequences. Overall the RM plots predicted a high quality model for the VP1 protein structure with over 95% of the residues in the most favored regions. RM plots of the Dunlop (1a) reference and our identified VP1 sequences indicated that the proteins share a high overall similarity but differ by specific amino acids in their 3-D conformation (Fig. 8c). For example, Ser-307 was conserved and located in the same position in all the three predicted RM plots, whereas Lys-353 was present in Dunlop (1a) and B12/A-62H (1a), but was replaced by Arg in B9/RU13 (IV). Similar differences were observed in other amino acids. The RM plot statistics of the VP1 protein for the reference Dunlop (1a) and two BKV subtypes (from two BKV+ patients) are given in Supplementary Table S6. A three-dimensional (3D) projection of the predicted structures using I-TASSER shows that the VP1 proteins from the different virus subtypes have different predicted residues in the binding sites, indicating the potential for functional effects on binding (Fig. 8d). A detailed description of the predicted binding sites for the VP1 protein are shown in Supplementary Figure S6.

We also observed polymorphisms in amino acids of the VP2 protein as predicted from Dunlop (1a) and the BKV genomes of B9/RU13 (IV) and B12/A-62H (1a), using ClustalW. The VP2 protein is a multifunctional structural protein in the BKV capsid and is involved in the binding to the host cell receptor together with VP1 protein. Overall, the predicted protein sequences were highly similar (Supplementary Figure S7). In another BKV genome, B10/A-47H (1b2), multiple sequence alignment for VP2 protein using ClustalW identified a nucleotide substitution at position 1257, which resulted in an amino acid change (His > Tyr) in the protein (Supplementary Figure S8 and Supplementary Table S7). RM plots of the VP2 protein of Dunlop (1a) strain, B9/RU13 (IV) and B12/A-62H (1a) BKV genomes predicted an overall good quality model, with over 95% of the residues in the most favored regions (Supplementary Figure S9a), and 3-D projection models of the VP2 proteins, revealed additional predicted binding sites in B9/RU13 (IV) and B12/A-62H (1a) (Supplementary Figures S9b and S10, Supplementary Table S8).

**Figure 9:**
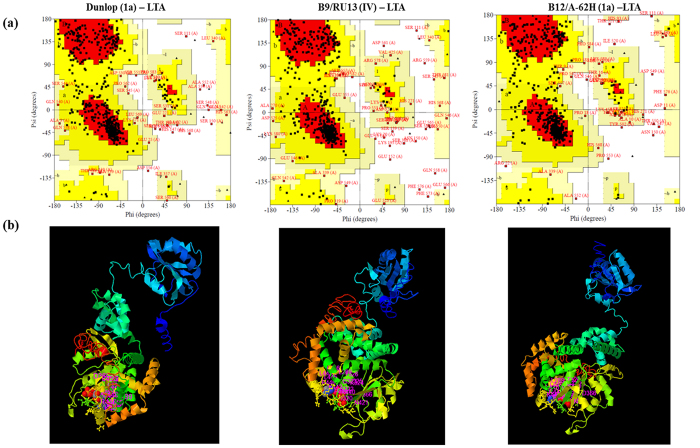
Analysis of Large T antigen protein of BK virus. (**a**) The Ramachandran plot for the phi-psi torsion angles for residues in the LTA protein of Dunlop (1a) (reference BKV subtype), B9/RU13 (IV) and B12/A-62H (1a) from BKV+ kidney transplant group. The coloring/shading on the plot represents the different regions described in Morris *et al*.^47^: the darkest areas (red) correspond to the “core” regions representing the most favorable combinations of phi-psi values. Over 90% of the residues in the “core” regions predicts a good quality structure model. The different regions on the Ramachandran plots are labelled as follows: A - Core alpha L - Core left-handed alpha a - Allowed alpha l - Allowed left-handed alpha ~a - Generous alpha ~l - Generous left-handed alpha B - Core beta p - Allowed epsilon b - Allowed beta ~p - Generous epsilon ~b - Generous beta. (**b**) Predicted 3D structure of the LTA protein.

Large T antigen (LTA) is a DNA binding protein of polyoma viruses that regulates immediate early gene expression following infection^28^. In addition, it can function as an oncogene and has been utilized to transform cell lines^29^. Based on our sequence alignments of LTA in Dunlop (1a), B9/RU13 (IV) and B12/A-62H (1a) genomes, we identified polymorphisms, some of which encoded non-conservative amino acid substitutions (Supplementary Figure S11), though the overall protein similarity score remained high (98–99%). For example, we identified a Leu > Pro substitution at position 1352 and a Ser > Gly substitution at position 4050 in B9/RU13 (IV) (Table 2). RM plots of LTA protein of Dunlop (1a) strain, B9/RU13 (IV) and B12/A-62H (1a) BKV genomes predicted an overall good quality model, with over 95% of the residues in the most favored regions (Fig. 9a, Supplementary Table S9), and 3-D projection model of the LTA proteins showed similar predicted binding sites in B9/RU13 (IV) and B12/A-62H (1a) in the ATP ligand (Fig. 9b and Supplementary Figure S12).

## Discussion

The development or reactivation of viral infections in immunosuppressed patients (such as transplant patients) causes significant morbidity and mortality. BKV has been linked to renal disease in organ transplant, and JCV has been associated with renal tubular epithelial infection and progressive multifocal leukoencephalopathy (PML). In this study, we used metagenome shotgun sequencing rather than the 16S rRNA amplicon based sequencing approach, as the latter is limited to the identification of bacteria classified by operational taxonomic units (OTUs) which impute genera. In contrast, the shotgun metagenomics strategy enables sequencing of nearly complete genomes and identifies all components of the microbiome including viruses, and even allows identification of virus subtypes and polymorphisms in specific amino acids in major virus proteins. Though the number of reads generated by the shotgun sequencing methods are generally not even across the samples and could potentially be biased due to an unequal number of sequences. However, the normalization procedures makes the relative abundance counts/ratios comparable among different samples. Previously, we have shown that in shotgun sequencing data a similar microbiome profile is detected in each sample irrespective of sampling depth in the same sample^30^. Similarly, other studies have also shown that even after random subsampling corresponding results were similar to those based on the original number of sequences^31^,^32^. Our analysis of the viruses in the urinary virome in kidney transplant patients provides several key conclusions. First, all of the patients analyzed in our study had detectable viruses in the urine (Supplementary Table S10). As expected, all of the patients classified as BKV+ (based on serum PCR) had high levels of virus in the urine. In addition, 9 of 15 patients classified as BKV− also had detectable polyoma virus in the urine with relative abundance ranging from 0.8 to 66.7% compared to the highest levels in BKV+ patients (Supplementary Table S1). The bulk of the polyoma virus reads were BKV. However, we detected significant levels of JCV ranging from 0.1 to 51.6% relative abundance in 5 patients (Supplementary Table S10). Interestingly, 3 of these patients had both JCV and BKV, whereas 2 patients had only JCV. We also detected *Anelloviridae* family viruses in multiple patients with the bulk being TTV (genus Alphatorquevirus). Ten patients had unclassified *Anelloviridae*, which was frequently abundant, but no patient had detectable Gammatorquevirus. For example, 87.9% of the total virus abundance in patient B16 was unclassified *Anelloviridae*. A recent study in lung transplant population noted a more complex population of Anelloviruses in broncho-alveolar lavage samples as compared to healthy controls^33^. A study by De Vlaminck *et al*., noted increased abundance of the *Anelloviridae* family upon immunosuppression, a trend also exhibited by polyoma viruses in immunosuppressed patients^34^. Our patient samples with high abundance of polyoma viruses were less abundant in Anelloviruses and vice versa. Both of these viruses are nuclear replicating viruses and possibly are competing against each other.

Interestingly, some viruses were detected in only a few or single patients. Three viruses that occurred in a subset of the patients were Mastadenovirus (positive in 11 patients), Sapovirus (positive in 4 patients) and Papillomavirus (positive in 5 patients). The genus Mastadenovirus encompasses 25 species including *Adenoviridae* that cause 2–5% of human respiratory infections, gastroenteritis and other human infections. Sapovirus includes the Sapporo virus, one of the two most common causes of acute viral gastroenteritis in adults. Whether urinary virus correlates with an acute infection remains to be determined. The Papillomaviruses were predominantly Betapapillomaviruses, with Alphapapillomavirus or Gammapapillomavirus, each only detected in one patient. The “unclassified” viruses match sequences in the database that have not been classified as a subtype. Lentivirus, a genus of the *Retroviridae* family that includes HIV and Visna virus, was present at 8.3% in a single patient. Similarly, Totivirus, which has fungi as natural hosts, was also detected at >8% in a single patient (B25) that, interestingly, also had detectable levels of *Candida tropicalis* and *Malassezia globose* (data not shown). There were also 11 patients with unclassified viruses which ranged in abundance up to 41.7% relative to other viruses, suggesting that the transplant population is infected with additional viruses that are not yet classified or well understood (Supplementary Table S1). Of note, some common viruses with clinical significance, such as Epstein-Barr virus, cytomegalovirus, and the herpes simplex viruses, were not detected in the urine, most likely because they are localized in other body compartments, e.g. the blood. Surprisingly, we also detected enterovirus B, a RNA virus, although in very low abundance (less than 0.001% of total virus reads) in 8 subjects. There have been reports that have described the integration of fragments of RNA viruses into host genomes. However, this occurrence of integration and maintenance of RNA virus genome fragments is primarily known to exist in insects and plants and has not previously been reported for enterovirus B, This phenomenon may provide defense against the future infection by the same or similar virus^35^,^36^,^37^,^38^.

A striking result in our study was the observation of multiple subtypes of the major urinary viruses BKV, JCV and TTV. For example, in total we detected 58, 31 and 108 different types of BKV, JCV, and TTV respectively, across the BKV+ and BKV− groups (Supplementary Table S5). Interestingly, the greatest number of subtypes detected in a single patient (B16) was 26 TTV subtypes. However, we also detected 11 subtypes of BKV in patient B5 and 7 subtypes of JCV in patient B19. On average, each patient was infected by multiple different subtypes of each virus including BKV, JCV and TTV (Supplementary Table S5). The pathological significance of the various subtypes is not well understood. In the BKV+ group, which all received the same immunosuppressive protocol, the virus subtypes did not correlate with the primary cause of ESRD. It has been reported that the pathogenesis of progressive multifocal leukoencephalopathy and renal epithelial infection is caused by different JCV subtypes. Our data indicate that multiple different virus subtypes in a single patient can coexist at relatively high abundance. It is tempting to speculate that a population of a virus composed of multiple subtypes could have an increased capacity to adapt to different stressors or environments and, thus, have a survival advantage. To investigate the potential functional effect of the virus subtypes we asked if the genetic polymorphisms were present in key viral proteins including VP1, VP2 and Large T antigen. Our analysis identified non-conservative amino acid substitutions in all 3 proteins. VP1, which is the major capsid protein, mediates virus attachment to the cell-surface sialic acid on gangliosides GT1b and GD1b which contain a terminal alpha (2–8)-linked sialic acid. Even minor changes in VP1 protein sequences can alter binding capacity and lead to large changes in infectivity^21^,^22^,^39^. Various substitutions have been reported in the VP1 protein in the literature and these could modulate pathogenicity. We identified non-conservative substitutions in VP1 in the binding site that attaches to the sialic acid on the cell surface receptor. VP2 protein is a minor capsid protein, and plays a role in host cell receptor binding together with VP1 and trafficking from the endoplasmic reticulum^40^,^41^. We identified non-conservative substitutions in VP2, such as, Ser > Asn, Arg > Gln in patient B9. Large T antigen, a DNA binding regulatory protein, similarly had non-conservative substitutions such as Leu > Pro and Ser > Gly. Our analysis of the predicted protein structure suggests these polymorphisms might affect function. The functions and pathogenicity of the various virus subtypes and polymorphisms that we identified in the transplant population merit further investigation relevant to graft survival and rejection in transplant patients.

Taken together, our results indicate that viruses were detected in all of our study patients, whether or not they were classified as BKV+ or BKV−. Also, many patients did not have a single dominant subtype, but rather had multiple virus subtypes. It will be important to determine the pathogenicity of the various virus subtypes in immunosuppressed patients.

## Material and Methods

### Study design, patient cohort and ethics statement

The objective of this study was to investigate the urinary virome in kidney transplant recipients with and without BK polyomavirus infection, using metagenome shotgun sequencing. The urine samples from the kidney transplant recipients with known comorbidities were analyzed. All Adult kidney transplant patients who received kidney transplants at the Washington University Medical Center, St. Louis, MO, were eligible for inclusion in the study. All patients were treated with a standard of care protocol. Four doses of rabbit anti-thymocyte globulin [Thymoglobulin (TMG) (1.5 mg/kg each), Sang-Stat Medical Corp., Fremont, CA] were administered; the first intra-operatively followed by doses on postoperative days 1–3. Dose adjustments were made for leukopenia or thrombocytopenia. Calcineurin inhibitors were instituted upon a brisk diuresis but no later than 4 days post-operatively. Cyclosporine was started at 8 mg/kg per day in two divided doses targeting 12 h whole blood trough levels of 250–300 ng/mL, which was monitored using a monoclonal fluorescent polarization immunoassay (FPIA) (Abbott Laboratories, Abbott Park, IL) during the first 3 months, then adjusted to 200–250 ng/mL. Alternatively, FK506 was initiated at 0.1 mg/kg per day divided in two doses adjusted to trough levels of 5–10 ng/mL by microparticle enzyme immunoassay (MEIA) (IMx, Abbott Laboratories, Abbott Park, IL). Methylprednisolone (7 mg/kg IV) was infused intra-operatively, then prednisone was started at 1 mg/kg per day, orally, postoperatively and tapered to 5–7.5 mg daily by the third month. All patients received prophylactic antibiotics (Bactrim). All urine samples were collected within the first year following transplantation during routine standard of care clinical follow-up. Clinical information including laboratory results, infections, and medications were collected for each subject at the time of each clinic visit. Comorbidities including Type 1 Diabetes Mellitus (T1DM), Type 2 Diabetes Mellitus (T2DM), hypertension (HTN), focal segmental glomerulosclerosis (FSGS), post-streptococcal glomerulonephritis, nephrocalcinosis (PSGN), polycystic kidney disease (PKD), reflux nephritis (RN), systemic lupus erythematous (lupus) and vasculitis were documented. The demographics of the transplant population are described in Table 1. Informed consent was obtained from all subjects. This study was approved by the Washington University School of Medicine Institutional Review Board, St. Louis, Missouri (IRB ID # 201102312, Protocol Number # 07-0430) and by the University of Illinois Institutional Review Board, Chicago, Illinois (IRB # 2014-1227), and the experimental methods were carried out in accordance with the approved guidelines.

### Enumeration of virus-like particles (VLP) in urine and metagenome DNA isolation and sequencing

Urine obtained by mid-stream clean catch was processed in sterile hoods in a positive pressure airflow room. Urine was centrifuged at 180 × *g* for 15 min at 4 °C to pellet and remove the mammalian cells, then the supernatant was centrifuged at 100,000 × g for 2 h at 4 °C in an ultra-centrifuge (Sorvall WX 80 Ultracentrifuge, Thermo Fisher Scientific Inc) to pellet the VLP and the microbial content, frozen at −80 °C. For the epifluorescence staining of the VLP, the pellet was re-suspended in 1 ml of 1 × PBS. stained with SyBr Gold (Invitrogen; Carlsbad, CA) for 10 min in the dark and visualized by epifluorescence microscopy as previously described^42^. Simultaneously another pellet was re-suspended with 100 μL 1 × PBS (Cellgro, Mediatech Inc.), and added 2 mM of MgCl_2_ and subjected to DNaseI treatment (2.5 U/ml) (Invitrogen) for 15 mins at 37 °C, and the reaction was stopped by adding 2 mM EDTA, and heating the contents for 10 min at 65 °C. The contents were subjected to enzymatic treatment by adding 800 μl AL Buffer (DNAeasy kit (Qiagen), Lysozyme (Fisher Bio Reagents) and enzyme solution (DNAeasy kit Handbook, Qiagen) for 2h at 37 °C. Proteinase K (80 μl) (DNAeasy kit, Qiagen) was added to the mixture and the DNA was eluted using elution columns following the DNAeasy kit (Qiagen) protocol. The purified DNA was quantified using a Qubit fluorometer (Life Technologies Corporation), and equal amount of DNA from all samples were mechanically fragmented using a Covaris S220 (Covaris Inc). The DNA libraries for sequencing were prepared using the NEBNext DNA library prep kit for Illumina (New England BioLabs Inc). The quality and quantity of all the DNA libraries were analyzed with a Bioanalyzer 2100 (Agilent Technologies) and Qubit fluorometer. Equal amount of all the DNA libraries were sequenced on Illumina HiSeq 2000 generating 50 base single end reads (BGI Americas). Control libraries were prepared by using 1 × PBS buffer instead of urine, and were subjected to the same DNA isolation protocol as for test urine samples. As expected there was no detectable DNA by Qubit analysis. The control DNA was then used to prepare metagenomic libraries, but no DNA was detected by Bioanalyzer analysis and thus not quantifiable. The control libraries were pooled and sequenced with the test libraries. After de-multiplexing of the sequence reads, no reads were assigned to the control libraries.

### Bioinformatics analyses, taxonomic, and statistical analyses

DNA sequence reads were quality trimmed and mapped against the reference human genome to remove human DNA sequences using the CLC Genomics Workbench with the default parameters (CLC Bio, Aarhus, Denmark). To investigate the urinary virome profile we used two approaches. First, the filtered non-human reads were analyzed using BLAST against the NCBI-nt database to ascertain the microbial taxonomy profile for each sample. The domain level taxonomy profile for bacteria, virus, eukaryotes and unclassified sequences was generated and relative abundance was calculated. From these total reads, the virus only reads were selected in-silico based on blast based taxonomy profile of each sample. All the reads identified from virus domain were selected in Fasta format and the respective taxonomy in csv format files. Second, to further investigate the nucleotide substitutions in whole genome and to determine the structure of major encoded proteins, the filtered non-human reads were assembled into contigs using CLC Genomics Workbench using default parameters. The contigs annotated to virus were identified using NBCI-nt BLAST. Virus counts for each sample were normalized using the RPKM (Reads per kb per million reads) and has been described in supplementary data. To verify the contig assembly, contigs were aligned to the reference virus genome sequences of BKV, JCV and TTV using the reported reference genomes as described previously^19^,^26^,^27^. A virus subtype specific genome alignment at nucleotide and amino acid level for virus proteins was performed using the ClustalW implementation, and visualized in Alignment Explorer in MEGA5^43^. Classification of the subtypes was based on >98% similarity with the reference genome sequence. Comparison among the groups were analyzed using GraphPad Prism (GraphPad Software, San Diego, CA, USA). Data were considered as statistically significant with *p* < 0.05 unless otherwise indicated.

### Phylogenetic tree analysis

Phylogenetic tree analysis was based on the complete genome sequences of the reference BK, JC and TT virus genomes and partial genome sequences of isolates identified in BKV+ and BKV− kidney transplant samples. The evolutionary history was inferred by the Maximum Likelihood method based on the Tamura-Nei model. The bootstrap consensus tree inferred from 100 replicates was assumed to represent the evolutionary history of the each taxa. Branches corresponding to partitions reproduced in less than 50% of the bootstrap replicates were collapsed. Each tree is drawn to scale, with branch lengths measured in the number of substitutions per site. Evolutionary analyses were conducted in MEGA5^43^.

### Protein structure prediction modeling

The amino acid sequences for major virus proteins of the Dunlop (1a) reference virus subtype was downloaded from NCBI reference genome. The assembled contigs which resulted in analysis of complete genomes of B9/RU13 (IV) and B12/A-62H (1a), were used for the open reading frame (ORF) prediction using the NCBI ORF Finder suite. The predicted ORFs for VP1, VP2, and Large T antigen were then confirmed using the BLASTp suite, and sequences were retrieved for further analysis. The protein structure and residue binding sites for Dunlop (1a) and other BKV sequences were predicted using the I-TASSER (Iterative Threading ASSEmbly Refinement) Suite for protein structure and function prediction^44^,^45^,^46^. I-TASSER is the most widely used web server to predict and build structural proteins from query sequences using the known structures as template from RCSB Protein Data Bank (PDB). The residue binding sites for Dunlop and other BKV sequences were predicted using the COFACTOR algorithm in I-TASSER Suite. COFACTOR is a structure-based method for biological function annotation of protein molecules, and provides possible binding ligands and the ligand-protein complex structures. The quality of the model is given by a C-score (range –5 to 2). The C-score is used in combination with the TM score (range 0–1) to obtain the best model. The quality of protein models obtained was further visualized and tested by Ramachandran (RM) plots^47^. For generating RM plots PROCHECK analysis implementation of PDBsum was used at EMBL-EBI web server^48^,^49^. The PROCHECK analyses provides the stereochemical quality of the predicted structure and highlights the regions of unusual geometry with an overall assessment of the structure.

## Additional Information

**How to cite this article**: Rani, A. *et al*. A diverse virome in kidney transplant patients contains multiple viral subtypes with distinct polymorphisms. *Sci. Rep.* **6**, 33327; doi: 10.1038/srep33327 (2016).

## Supplementary Material

Supplementary Information

## Figures and Tables

**Table 1:** Characteristics of kidney transplant patient’s samples.

**Group**	**Sample**	**Gender**	**Age**	**Ethnicity**	**Clinical Diagnosis**	**Calcineurin Inhibitors**	**BK Viruria**	**BK Viremia**
BKV+								
	B3	M	59	Caucasian	T1DM	FK	Yes	Yes
	B4							
	B5	M	76	African-American	T2DM	FK	Yes	Yes
	B6							
	B7	M	31	Caucasian	PSGN	FK	Yes	Yes
	B8							
	B9	M	47	Caucasian	PKD	CYA	Yes	Yes
	B10	M	59	Caucasian	HTN	FK	Yes	Yes
	B11							
	B12	M	65	Caucasian	HTN	FK	Yes	Yes
	B13							
	B15	M	66	Caucasian	T2DM	FK	Yes	Yes
BKV−	B16	M	52	African-American	HTN	CYA	No	No
	B17	F	62	African-American	T1DM	FK	No	No
	B18	F	40	Caucasian	Vasculitis	CYA	No	No
	B19	F	45	Asian	FSGS	CYA	No	No
	B20	M	51	Caucasian	T2DM	FK	No	No
	B21	M	41	Caucasian	HTN	FK	Yes	No
	B22	F	51	Caucasian	Lupus	FK	No	No
	B23	F	51	Caucasian	T1DM	CYA	Yes	No
	B24	F	46	Caucasian	RN	FK	Yes	No
	B25	M	54	Caucasian	HTN	FK	No	No
	B26	F	74	Caucasian	HTN	FK	No	No
	B27	M	46	Caucasian	HTN	FK	No	No
	B28	M	54	Caucasian	PKD	FK	No	No
	B29	F	32	Caucasian	T1DM	FK	Yes	No
	B30	M	68	Caucasian	Nephrocalcinosis	FK	No	No

Demographic data including sample number, gender, age, ethnicity, ESRD cause, calcineurin inhibitor agent. All patients received Thymoglobulin for induction and a calcineurin inhibitor along with mycophenolate and prednisone for maintenance. Samples were collected during the first 12 months post transplantation. T1DM (Type 1 Diabetes Mellitus); T2DM (Type 2 Diabetes Mellitus); HTN (Hypertension); FSGS (Focal Segmental Glomerulosclerosis); PSGN (Post Streptococcal Glomerulonephritis); PKD (Polycystic Kidney Disease); RN (Reflux Nephropathy).

**Table 2:** Nucleotide substitution in Large T Antigen.

**Reference genome/Sample**	**Subtype/Subgroup**	**Dunlop nucleotide position**	**Dunlop amino acid position**	**Nucleotide substitutions**	**Translated protein code**
Dunlop	Ia	3997	1335	AAG	K (Lys)
SJH-LG-309	Ib1			AAG	K (Lys)
B9	Ib1			A**G**G	R (Arg)
Dunlop	Ia	4021	1343	GCT	A (Ala)
SJH-LG-309	Ib1			GCT	A (Ala)
B9	Ib1			G**T**T	V (Val)
Dunlop	Ia	4024	1344	CTT	L (Leu)
SJH-LG-309	Ib1			CTT	L (Leu)
B9	Ib1			C**C**T	P (Pro)
Dunlop	Ia	4050	1353	AAG	S (Ser)
SJH-LG-309	Ib1			AAG	S (Ser)
B9	Ib1			A**G**G	R (Arg)

Note: Nucleotide substitutions are indicated in bold and underline.
