# Supplementary material for: A diverse virome in kidney transplant patients contains multiple viral subtypes with distinct polymorphisms
Source: Sci Rep. 2016 Sep 16;6:33327. doi: 10.1038/srep33327 (PMC5025891; doi:10.1038/srep33327)
Supplement: Supplementary Information [file srep33327-s1.pdf]

SUPPLEMENTARY DATA

A diverse virome in kidney transplant patients contains multiple viral subtypes with distinct polymorphisms

Asha Rani[1†], Ravi Ranjan[1†], Halvor S. McGee[1], Ahmed Metwally[1,2], Zahraa Hajjiri[1], Daniel C. Brennan[3], Patricia W. Finn[1], David L. Perkins[1,2,4,*]

[1]Department of Medicine, University of Illinois, Chicago, IL 60612 USA

[2]Department of Bioengineering, University of Illinois, Chicago, IL 60612 USA

[3]Division of Renal Diseases, Washington University School of Medicine, St. Louis, MO 63110 USA

[4]Department of Surgery, University of Illinois, Chicago, IL 60612 USA

[*]**Corresponding author:**

David Perkins, MD, PhD

University of Illinois at Chicago

Department of Medicine, MC 787

840 S Wood Street, Suite 1020N CSB

Chicago IL 60612 USA

Email: perkinsd@uic.edu , Phone: 312-413-3382, Fax: 312-355-0499

[†]These authors contributed equally and considered as co-first authors.

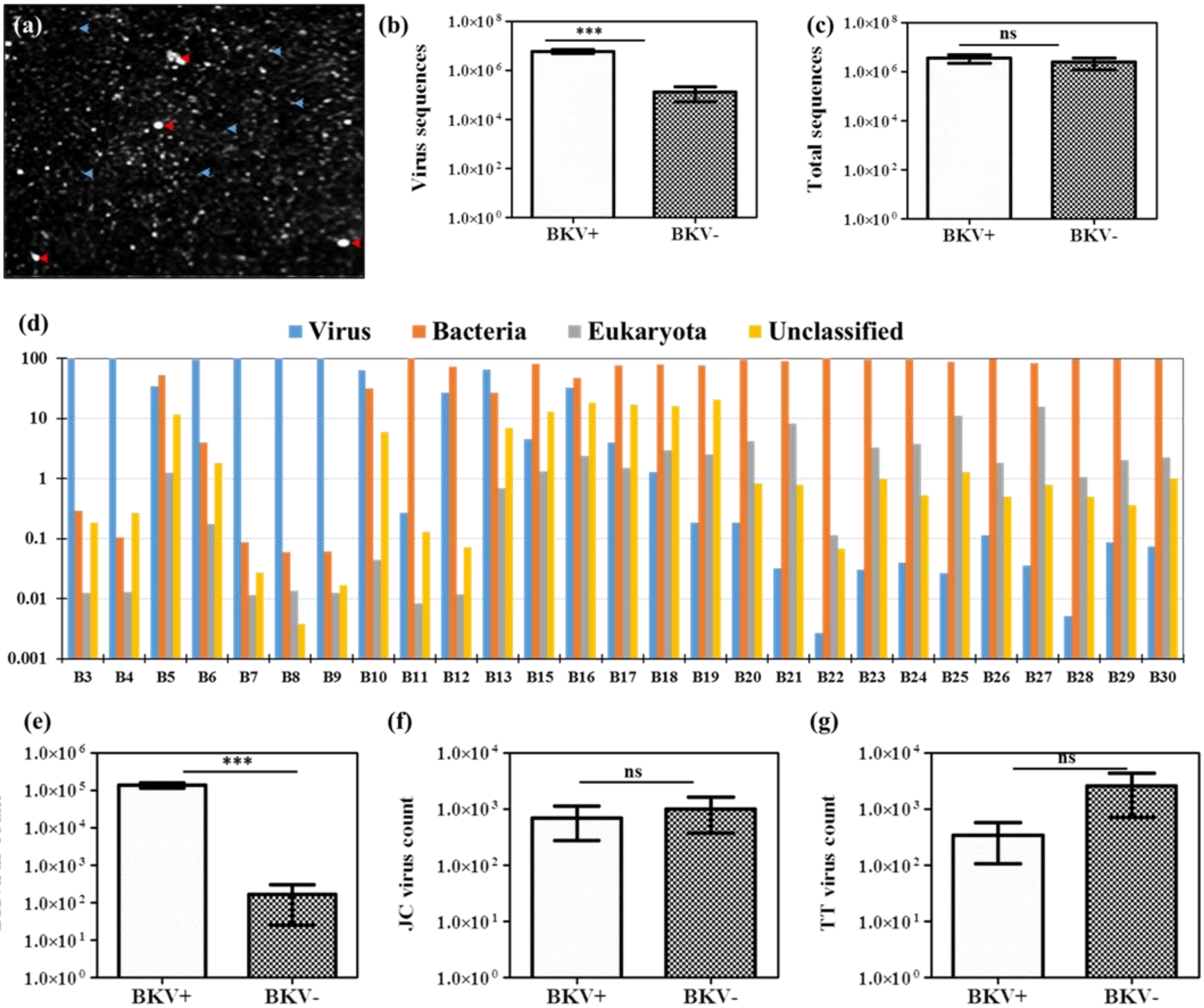

Supplementary Figure S1. Virus-like particles (VLP) identification and shotgun metagenomics. (a) VLP was detected in urine of the transplant patients by SyBr Gold staining. The VLP appear as dim pinpoints (denoted by blue arrows) and the microbial cells which are larger and brighter (denoted by red arrows). (b) The sequences were filtered to remove human reads. The total number of virus sequences identified in each group, are significantly different. The difference was computed using Welch *t*-test, $^{***}p<0.001$. (c) Total number of sequences (non-human and non-virus) identified after filtering for virus reads in BKV+ and BKV- samples. No significant (ns) difference was observed in each group. (d) Bar graph representation for distribution of total number of virus, bacteria, eukaryotic and unclassified reads among BKV+ and BKV- samples. (e) BKV, (f) JCV and (g) TTV counts in BKV+ and BKV- groups. BKV was dominant in the BKV+ group and was statistically significant among the groups. The difference was computed using Welch *t*-test, ***$p<0.001$. There was no significant (ns) difference among the JCV and TT virus counts, in both the groups, though virus count for both JC and TTV were slightly higher in BKV- groups.

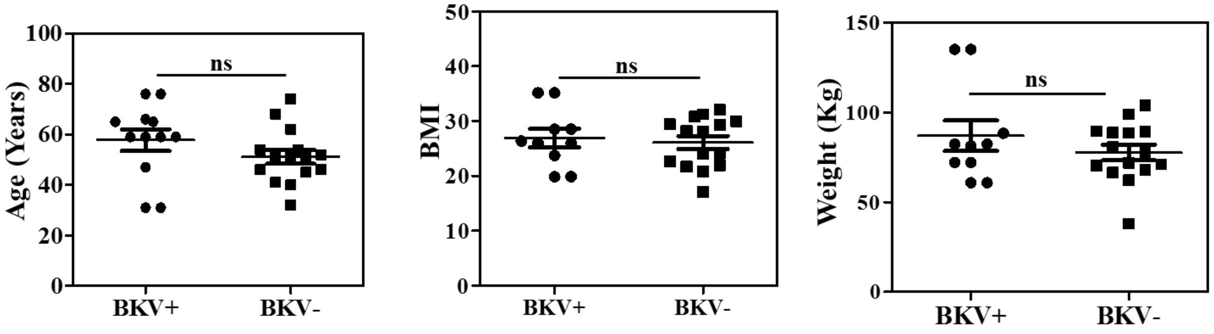

Supplementary Figure S2. BKV+ and BKV- group patient's demographics. No correlation was observed between patient's demographics and clinical diagnosis with the status of viremia or viruria in BKV+ and BKV- group. The difference was computed using Welch *t*-test.

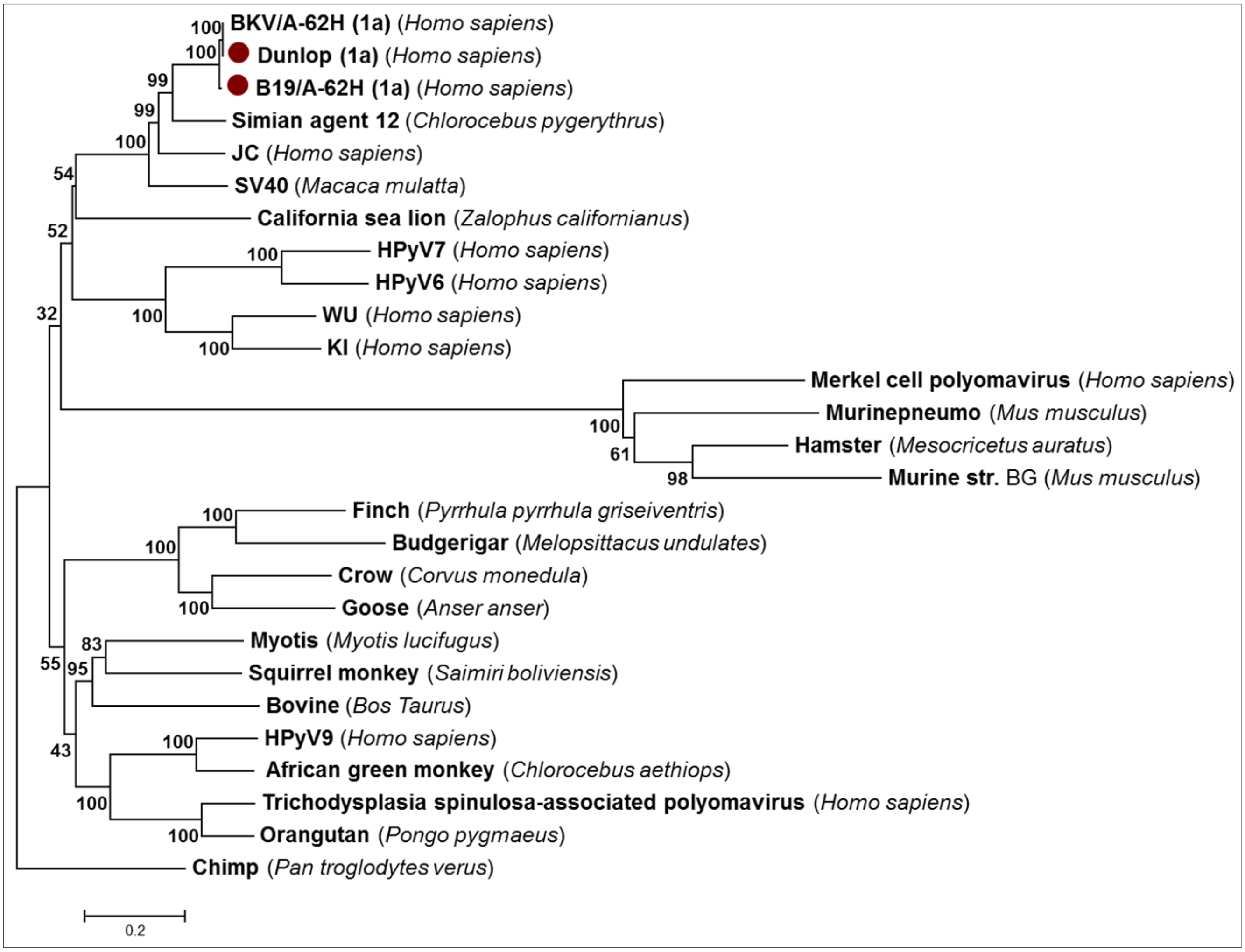

Supplementary Figure S3. Species specific phylogenetic analysis for BK virus. Phylogenetic tree analysis is based on the whole genome sequences of the 26 species-specific distinct BKV strains and sequences from this study [e.g. the B19/A-62H (1a) sequence]. Our sequence clustered adjacent to the human BKV Dunlop (1a) reference strain described in this study. The evolutionary history was inferred by using the Maximum Likelihood method based on the Tamura-Nei model. The bootstrap consensus tree inferred from 100 replicates is taken to represent the evolutionary history of the taxa analyzed. The tree is drawn to scale, with branch lengths measured in the number of substitutions per site.

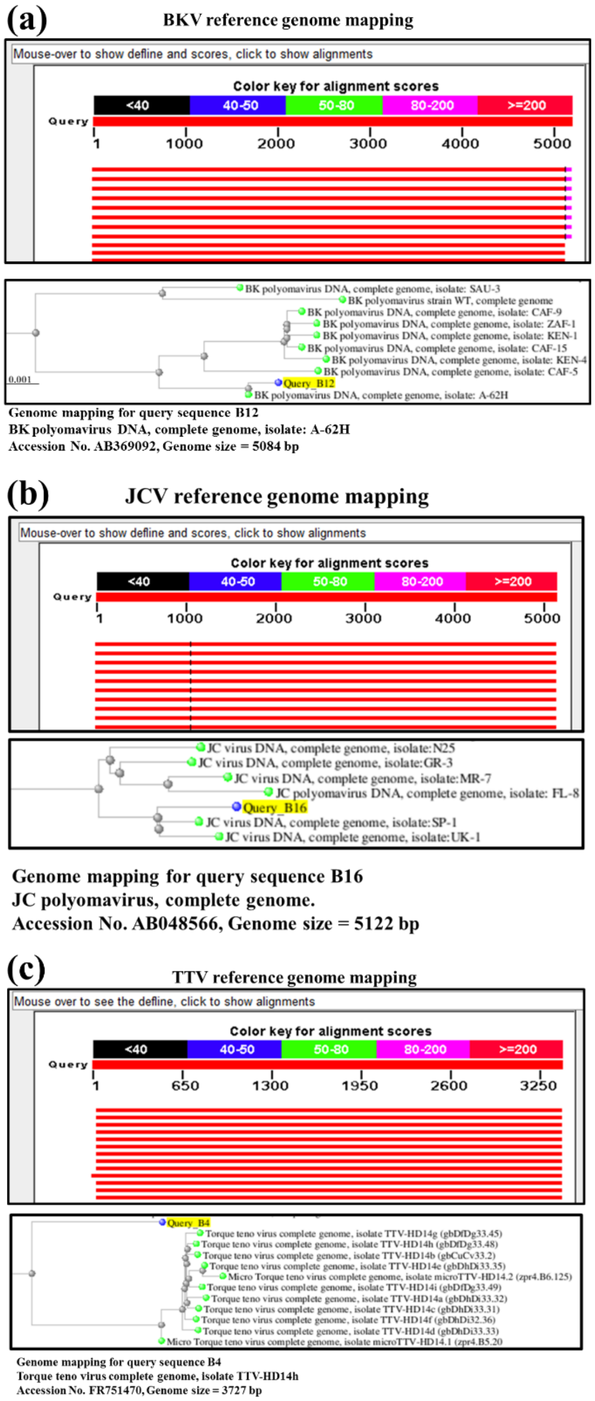

Supplementary Figure S4. (a) Reference genome mapping for BK virus (a), JC virus and (b), TT virus (c). The query sequences (highlighted in yellow in phylogenetic view) were mapped to reference virus genome sequences to identify virus specific subtype. A blast alignment and phylogenetic view of the mapping is shown for each query sequence against a reference subtype using NCBI webserver.

```
Dunlop (1a)_VP1        MKMAPTKRKGECPGAAPKKPKEPVQVPKLLIKGGVEVLEVKTGVDAITEVECFLNPEMGD
B12/A-62H (1a)_VP1     MKMAPTKRKGECPGAAPKKPKEPVQVPKLLIKGGVEVLEVKTGVDAITEVECFLNPEMGD
B9/RU13 (IV)_VP1       MKMAPTKRKGECPGAAPKKPKEPVQVPKLLIKGGVEVLEVKTGVDAITEVECFLNPEMGD
                       ************************************************************

Dunlop (1a)_VP1        PDENLRGFSLKLSAENDFSSDSPERKMLPCYSTARIPLPNLNEDLTCGNLLMWEAVTVQT
B12/A-62H (1a)_VP1     PDENLRGFSLKLSAENNFSSDSPERKMLPCYSTARIPLPNLNEDLTCGNLLMWEAVTVQT
B9/RU13 (IV)_VP1       PDNDLRGYSLRLTAETAFDSDSPDRKMLPCYSTARIPLPNLNEDLTCGNLLMWEAVTVKT
                       **::***:**:*:**. *.****:**********************************:*

Dunlop (1a)_VP1        EVIGITSMLNLHAGSQKVHEHGGGKPIQGSNFHFFAVGGEPLEMQGVLMNYRSKYPDGTI
B12/A-62H (1a)_VP1     EVIGITSMLNLHAGSQKVHEHGGGKPIQGSNFHFFAVGGDPLEMQGVLMNYRTKYPDGTI
B9/RU13 (IV)_VP1       EVIGITSMLNLHAGSQKVHENGGGKPIQGSNFHFFAVGGDPLEMQGVLMNYRTKYPEGTV
                       ********************:******************:************:***:**:

Dunlop (1a)_VP1        TPKNPTAQSQVMNTDHKAYLDKNNAYPVECWVPDPSRNENARYFGTFTGGENVPPVLHVT
B12/A-62H (1a)_VP1     TPKNPTAQSQVMNTDHKAYLDKNNAYPVECWVPDPSRNENTRYFGTFTGGENVPPVLHVT
B9/RU13 (IV)_VP1       TPKNPTAQSQVMNTDHKAYLDKNNAYPVECWIPDPSRNENTRYFGTYTGGENVPPVLHVT
                       *******************************:********:*****:*************

Dunlop (1a)_VP1        NTATTVLLDEQGVGPLCKADSLYVSAADICGLFTNSSGTQQWRGLARYFKIRLRKRSVKN
B12/A-62H (1a)_VP1     NTATTVLLDEQGVGPLCKADSLYVSAADICGLFTNSSGTQQWRGLARYFKIRLRKRSVKN
B9/RU13 (IV)_VP1       NTATTVLLDEQGVGPLCKADSLYVSAADICGLFTNSSGTQQWRGLPRYFKIRLRKRSVKN
                       *********************************************.**************

Dunlop (1a)_VP1        PYPISFLLSDLINRRTQRVDGQPMYGMESQVEEVRVFDGTERLPGDPDMIRYIDKQGQLQ
B12/A-62H (1a)_VP1     PYPISFLLSDLINRRTQRVDGQPMYGMESQVEEVRVFDGTERLPGDPDMIRYIDKQGQLQ
B9/RU13 (IV)_VP1       PYPISFLLSDLINRRTQRVDGQPMYGMESQVEEVRVFDGTEQLPGDPDMIRYIDRQGQLQ
                       *****************************************:************:*****

Dunlop (1a)_VP1        TKML
B12/A-62H (1a)_VP1     TKML
B9/RU13 (IV)_VP1       TKMV
                       ***:
```

Note

```
Sequence 1: Dunlop (1a)_VP1      364 aa
Sequence 2: B9/RU13 (IV)_VP1     364 aa
Sequence 3: B12/A-62H (1a)_VP1   364 aa

Alignment Score
Sequences (1:2) Aligned. Score: 93.956
Sequences (1:3) Aligned. Score: 98.9011
Sequences (2:3) Aligned. Score: 94.7802
```

Supplementary Figure S5. Multiple sequence alignment using ClustalW for large VP1 protein. Overall protein similarity score was high (more than 93%), however, there were amino acid substitutions and are highlighted in box.

VP1 protein

BKV Dunlop (1a)

Click to view	Rank	Cscore^{LB}	PDB Hit	TM-score	RMSD^{a}	IDEN^{a}	Cov.	BS-score	Lig. Name	Download Complex	Predicted binding site residues
◉	1	0.70	3bwrC	0.698	1.44	0.778	0.715	1.05	SIA	Download	62,68,69,279
○	2	0.22	3bwrA	0.697	1.44	0.772	0.715	1.04	Mul.Part	Download	69,70,85

B9/RU13 (IV)

Click to view	Rank	Cscore^{LB}	PDB Hit	TM-score	RMSD^{a}	IDEN^{a}	Cov.	BS-score	Lig. Name	Download Complex	Predicted binding site residues
○	1	0.73	3bwrC	0.696	1.44	0.797	0.713	1.58	Mul.Part	Download	68,69,70,71,85,275,277
○	2	0.72	3bwrA	0.695	1.44	0.794	0.713	1.44	SIA	Download	63,66,68,69,273,275,277,279

B12/A-62H (1a)

Click to view	Rank	Cscore^{LB}	PDB Hit	TM-score	RMSD^{a}	IDEN^{a}	Cov.	BS-score	Lig. Name	Download Complex	Predicted binding site residues
◉	1	0.71	3bwrC	0.698	1.46	0.782	0.715	1.20	SIA	Download	63,68,69,273,277,279
○	2	0.22	3bwrE	0.698	1.46	0.780	0.715	0.97	Mul.Part	Download	69,70,71,85

Cscore is the confidence score of predicted binding site. It ranges in between [0-1]; a higher score indicates a more reliable ligand-binding site prediction. **BS-score** is a measure of local similarity (sequence & structure) between template binding site and predicted binding site in the query structure. A BS-score >1 reflects a significant local match between the predicted and template binding site. **TM-score** is a measure of global structural similarity between query and template protein. **RMSD** the RMSD between residues that are structurally aligned by TM-align. **IDEN** is the percentage sequence identity in the structurally aligned region. **Cov.** represents the coverage of global structural alignment and is equal to the number of structurally aligned residues divided by length of the query protein. The detailed description can be viewed at http://zhanglab.ccmb.med.umich.edu/COFACTOR/

Supplementary Figure S6. Predicted residue binding sites for VP1 protein in reference Dunlop (1a) strain and B9/RU13 (IV) and B12/A-62H (1a) from two BKV+ patient's described in this study. Predicted binding sites for ligand sialic acid (SIA) are highlighted with circle. The residue binding sites for Dunlop and other BKV sequences were predicted using the COFACTOR algorithm in I-TASSER Suite.

```
BKV Dunlop (1a)-VP2 MGAALALLGDLVASVSEAAAATGFSVAEIAAGEAAAAIEVQIASLATVEGITSTSEAIAA
B12/A-62H (1a)-VP2  MGAALALLGDLVASVSEAAAATGFSVAEIAAGEAAAAIEVQIASLATVEGITSTSEAIAA
B9/RU13 (IV)_VP2    MGAALALLGDLVASVSEAAAATGFSVAEIAAGEAAAAIEVQIASLATVEGITSTSEAIAA
                    ************************************************************

BKV Dunlop (1a)-VP2 IGLTPQTYAVIAGAPGAIAGFAALIQTVSGISSLAQVGYKFFDDWDHKVSTVGLYQQSGM
B12/A-62H (1a)-VP2  IGLTPQTYAVIAGAPGAIAGFAALIQTVSGISSLAQVGYRFFSDWDHKVSTVGLYQQSGM
B9/RU13 (IV)_VP2    IGLTPQTYAVIAGAPGAIAGFAALIQTVTGISSLAQVGYRFFSDWDHKVSTVGLYQQSGM
                    ****************************:**********:**.*****************

BKV Dunlop (1a)-VP2 ALELFNPDEYYDILFPGVNTFVNNIQYLDPRHWGPSLFATISQALWHVIRDDIPSITSQE
B12/A-62H (1a)-VP2  ALELFNPDEYYDILFPGVNTFVNNIQYLDPRHWGPSLFATISQALWHVIRDDIPSITSQE
B9/RU13 (IV)_VP2    ALELFNPDEYYDILFPGVNTFVNNIQYLDPRHWGPSLFATISQALWHVIRDDIPAITSQE
                    ******************************************************:*****

BKV Dunlop (1a)-VP2 LQRRTERFFRDSLARFLEETTWTIVNAPINFYNYIQQYYSDLSPIRPSMVRQVAEREGTR
B12/A-62H (1a)-VP2  LQRRTERFFRDSLARFLEETTWTIVNAPINFYNYIQQYYSDLSPIRPSMVRQVAEREGTR
B9/RU13 (IV)_VP2    LQRRTERFFRDSLARFLEETTWTIVNAPVNFYNYIQDYYSNLSPIRPSMVRQVAEREGTQ
                    ****************************:*******:***:******************:

BKV Dunlop (1a)-VP2 VHFGHTYSIDDADSIEEVTQRMDLRNQQSVHSGEFIEKTIAPGGANQRTAPQWMLPLLLG
B12/A-62H (1a)-VP2  VHFGHTYSIDDADSIEEVTQRMDLRNQQSVHSGEFIEKTIAPGGANQRTAPQWMLPLLLG
B9/RU13 (IV)_VP2    VNFGHTYRIDDADSIQEVTQRMELRNKENVHSGEFIEKTIAPGGANQRTAPQWMLPLLLG
                    *:***** *******:******:***::.*******************************

BKV Dunlop (1a)-VP2 LYGTVTPALEAYEDGPNQKKRRVSRGSSQKAKGTRASAKTTNKRRSRSSRS
B12/A-62H (1a)-VP2  LYGTVTPALEAYEDGPNQKKRRVSRGSSQKAKGTRASAKTTNKRRSRSSRS
B9/RU13 (IV)_VP2    LYGTVTPALEAYEDGPNQKKRRVSRGSSQKAKGTRASAKTTNKRRSRSSRS
                    ***************************************************
```

Note

Sequence 1: BKV Dunlop (1a)-VP2 351 aa
Sequence 2: B9/RU13 (IV)_VP2 351 aa
Sequence 3: B12/A-62H (1a)-VP2 351 aa

Alignment Score:
Sequences (1:2) Aligned. Score: 95.7265
Sequences (1:3) Aligned. Score: 99.4302
Sequences (2:3) Aligned. Score: 96.2963

Supplementary Figure S7. Multiple sequence alignment using ClustalW for VP2 protein. Overall protein similarity score was high (more than 95%). The amino acid substitutions and are highlighted in box.

Virus subtype		*	*	*	*	*	*	*	*	*	*	*	*	*	
1. DUNLOP (Ia)	T	A	T	A	A	T	T	A	T	A	T	T	C	A	A
2. A-62H (Ia)	T	A	T	A	A	T	T	A	T	A	T	T	C	A	A
3. SJH-LG-152 (Ib1)	T	A	T	A	A	T	T	A	T	A	T	T	C	A	A
4. SJH-LG-306 (Ib1)	T	A	T	A	A	T	T	A	T	A	T	T	C	A	A
5. SJH-LG-309 (Ib1)	T	A	T	A	A	T	T	A	T	A	T	T	C	A	A
6. LAB-27 (Ib1)	T	A	T	A	A	T	T	A	T	A	T	T	C	A	A
7. A-68H (Ib1)	T	A	T	A	A	T	T	A	T	A	T	T	C	A	A
8. J2B-2 (Ib1)	T	A	T	A	A	T	T	A	T	A	T	T	C	A	A
9. A-43H (Ib1)	T	A	T	A	A	T	T	A	T	A	T	T	C	A	A
10. J3B-3 (Ib2)	T	A	T	A	A	T	T	A	T	A	T	T	C	A	A
11. A-47H (Ib2)	T	A	T	A	A	T	T	A	T	A	T	T	C	A	A
12. SJH-LG-308 (Ib2)	T	A	T	A	A	T	T	A	T	A	T	T	C	A	A
13. RU13 (IV)	T	A	T	A	A	T	T	A	T	A	T	T	C	A	G
14. RU15 (IV)	T	A	T	A	A	T	T	A	T	A	T	T	C	A	G
15. LAB-33 (IV)	T	A	T	A	A	T	T	A	T	A	T	T	C	A	G
16. B3/J2B-2 (Ib1)	T	A	T	A	A	T	T	A	T	A	T	T	C	A	A
17. B4/A-47H (Ib2)	T	A	T	A	A	T	T	A	T	A	T	T	C	A	A
18. B7/LAB-27 (Ib1)	T	A	T	A	A	T	T	A	T	A	T	T	C	A	A
19. B8/LAB-27 (Ib1)	T	A	T	A	A	T	T	A	T	A	T	T	C	A	A
20. B9/RU13 (IV)	T	A	T	A	A	T	T	A	T	A	T	T	C	A	G
21. B10/A-47H (Ib2)	C	A	T	A	A	T	T	A	T	A	T	T	C	A	A
22. B11/A-47H (Ib2)	T	A	T	A	A	T	T	A	T	A	T	T	C	A	A
23. B12/A-62H (Ia)	T	A	T	A	A	T	T	A	T	A	T	T	C	A	A
24. B16/SJH-LG-309 (Ib1)	T	A	T	A	A	T	T	A	T	A	T	T	C	A	A
25. B18/A-62H (Ia)	T	A	T	A	A	T	T	A	T	A	T	T	C	A	A
26. B19/J2B-2 (Ib1)	T	A	T	A	A	T	T	A	T	A	T	T	C	A	A

Nucleotide position in BK virus genome (Dunlop) 1257. VP2 protein region. Note for variation in B10 sample.

Species/Abbrv		*	*	*	*
1. DUNLOP (Ia)	Y	N	Y	I	Q
2. A-62H (Ia)	Y	N	Y	I	Q
3. SJH-LG-152 (Ib1)	Y	N	Y	I	Q
4. SJH-LG-306 (Ib1)	Y	N	Y	I	Q
5. SJH-LG-309 (Ib1)	Y	N	Y	I	Q
6. LAB-27 (Ib1)	Y	N	Y	I	Q
7. A-68H (Ib1)	Y	N	Y	I	Q
8. J2B-2 (Ib1)	Y	N	Y	I	Q
9. A-43H (Ib1)	Y	N	Y	I	Q
10. J3B-3 (Ib2)	Y	N	Y	I	Q
11. A-47H (Ib2)	Y	N	Y	I	Q
12. SJH-LG-308 (Ib2)	Y	N	Y	I	Q
13. RU13 (IV)	Y	N	Y	I	Q
14. RU15 (IV)	Y	N	Y	I	Q
15. LAB-33 (IV)	Y	N	Y	I	Q
16. B3/J2B-2 (Ib1)	Y	N	Y	I	Q
17. B4/A-47H (Ib2)	Y	N	Y	I	Q
18. B7/LAB-27 (Ib1)	Y	N	Y	I	Q
19. B8/LAB-27 (Ib1)	Y	N	Y	I	Q
20. B9/RU13 (IV)	Y	N	Y	I	Q
21. B10/A-47H (Ib2)	H	N	Y	I	Q
22. B11/A-47H (Ib2)	Y	N	Y	I	Q
23. B12/A-62H (Ia)	Y	N	Y	I	Q
24. B16/SJH-LG-309 (Ib1)	Y	N	Y	I	Q
25. B18/A-62H (Ia)	Y	N	Y	I	Q
26. B19/J2B-2 (Ib1)	Y	N	Y	I	Q

Amino acid substitution in VP protein. H: Histidine, Y: Tyrosine.

Supplementary Figure S8. Multiple sequence alignment using ClustalW for VP2 protein. Multiple sequence alignment revealed a nucleotide substitution at position 1257 in BKV genome B10/A-47H (1b2), which resulted in an amino acid change from His>Tyr in the protein.

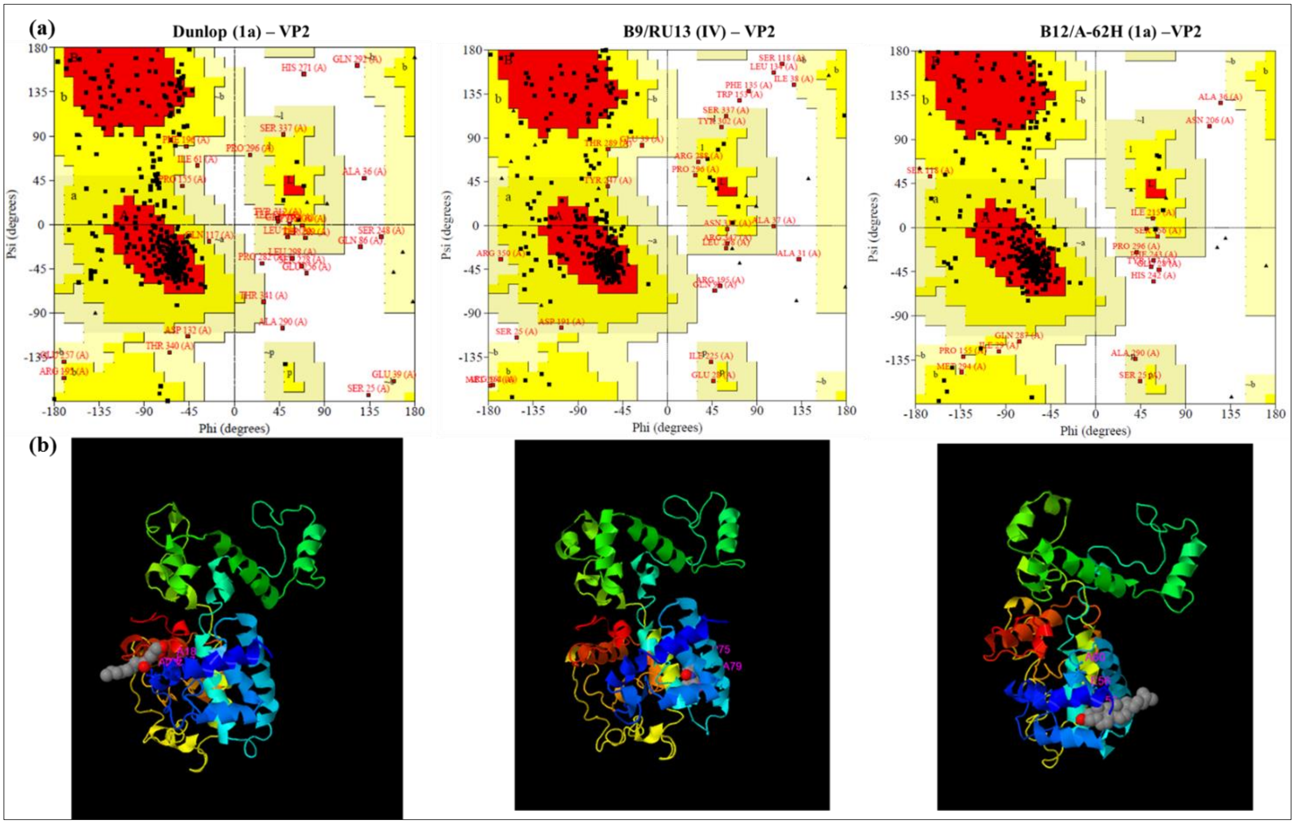

Supplementary Figure S9. Analysis of VP2 protein of BK virus. (a) The Ramachandran plot for the phi-psi torsion angles for residues in the VP2 protein of Dunlop (1a) (reference BKV subtype), B9/RU13 (IV) and B12/A-62H (1a) from BKV+ kidney transplant group. The coloring/shading on the plot represents the different regions described in Morris *et al.* (1992): the darkest areas (red) correspond to the "core" regions representing the most favorable combinations of phi-psi values. Over 90% of the residues in the "core" regions predicts a good quality structure model. The different regions on the Ramachandran plots are labelled as follows: A - Core alpha L - Core left-handed alpha a - Allowed alpha l - Allowed left-handed alpha ~a - Generous alpha ~l - Generous left-handed alpha B - Core beta p - Allowed epsilon b - Allowed beta ~p - Generous epsilon ~b - Generous beta. (b) Predicted 3D structure of the VP2 protein.

VP2 Protein

BKV Dunlop (1a)

Click to view	Rank	Cscore^{LB}	PDB Hit	TM-score	RMSD^{a}	IDEN^{a}	Cov.	BS-score	Lig. Name	Download Complex	Predicted binding site residues
○	1	0.01	3oe6A	0.329	4.14	0.046	0.393	0.65	OLC	Download	17,18,21,22

B9/RU13 (IV)

Click to view	Rank	Cscore^{LB}	PDB Hit	TM-score	RMSD^{a}	IDEN^{a}	Cov.	BS-score	Lig. Name	Download Complex	Predicted binding site residues
○	1	0.01	2q9eB	0.282	3.13	0.094	0.333	0.62	MTN	Download	75,79,91
○	2	0.01	1xepA	0.277	3.11	0.096	0.328	0.47	CAQ	Download	77,80,81,91
○	3	0.01	1owyA	0.278	3.09	0.096	0.328	0.47	PRY	Download	75,79,84,88,91,94

B12/A-62H (1a)

Click to view	Rank	Cscore^{LB}	PDB Hit	TM-score	RMSD^{a}	IDEN^{a}	Cov.	BS-score	Lig. Name	Download Complex	Predicted binding site residues
○	1	0.01	3ny9A	0.389	6.16	0.079	0.610	0.40	CLR	Download	52,56,60
○	2	0.01	2pflA	0.331	6.87	0.047	0.556	0.40	NA	Download	19,22,23,24
○	3	0.01	3oduB	0.338	5.68	0.069	0.470	0.41	OLC	Download	52,58,62

Supplementary Figure S10. Predicted residue binding sites for VP2 protein in reference Dunlop (1a) strain and B9/RU13 (IV) and B12/A-62H (1a) from two BKV+ patient's described in this study. The residue binding sites for Dunlop (1a) and other BKV sequences were predicted using the COFACTOR algorithm in I-TASSER Suite.

```
BKV Dunlop (1a)-LTA   MFASDEEATADSQHSTPPKKKRKVEDPKDFPSDLHQFLSQAVFSNRTLACFAVYTTKEKA
B12/A-62H (1a)-LTA    MFASDEEATADSQHSTPPKKKRKVEDPKDFPSDLHQFLSQAVFSNRTLACFAVYTTKEKA
B9/RU13 (IV)_LTA      MFASDEEATADSQHSTPPKKKRKVEDPKDFPSDLHQFLSQAVFSNRTLACFAVYTTKEKA
                      ************************************************************

BKV Dunlop (1a)-LTA   QILYKKLMEKYSVTFISRHMCAGHNIIFFLTPHRHRVSAINNFCQKLCTFSFLICKGVNK
B12/A-62H (1a)-LTA    QILYKKLMEKYSVTFISRHMCAGHNIIFFLTPHRHRVSAINNFCQKLCTFSFLICKGVNK
B9/RU13 (IV)_LTA      LILYKKLMEKYSVTFISRHMCAGHNIIFFLTPHRHRVSAINNFCQKLCTFSFLICKGVNK
                       ***********************************************************

BKV Dunlop (1a)-LTA   EYLLYSALTRDPYHTIEESIQGGLKEHDESPEEPEETKQVSWKLITEYAVETKCEDVFLL
B12/A-62H (1a)-LTA    EYLLYSALTREPYHTIEESIQGGLKEHDENPEEPEETKQVSWKLITEYAVETKCEDVFLL
B9/RU13 (IV)_LTA      EYLLYSALTRDPYYIIEESIQGGLKEHDENPEEPEETKQVSWKLITEYAVETKCEDVFLL
                      **********:**: **************.******************************

BKV Dunlop (1a)-LTA   LGMYLEFQYNVEECKKCQKKDQPYHFKYHEKHFANAIIFAESKNQKSICQQAVDTVLAKK
B12/A-62H (1a)-LTA    LGMYLEFQYNVEECKKCQKKDQPYHFKYHEKHFANAIIFAESKNQKSICQQAVDTVLAKK
B9/RU13 (IV)_LTA      LGMYLEFQYNVEECKKCQKKDQPYHFKYHEKHFANATIFAESKNQKSICQQAVDTVLAKK
                      ************************************ ***********************

BKV Dunlop (1a)-LTA   RVDTLHMTREEMLTERFNHILDKMDLIFGAHGNAVLEQYMAGVAWLHCLLPKMDSVIFDF
B12/A-62H (1a)-LTA    RVDTLHMTREEMLTERFNHILDKMDLIFGAHGNAVLEQYMAGVAWLHCLLPKMDSVIFDF
B9/RU13 (IV)_LTA      RVDTLHMTREEMLTDRFNHILDKMDLIFGAHGNAVLEQYMAGVAWLHCLLPKMDSVIFDF
                      **************:*********************************************

BKV Dunlop (1a)-LTA   LHCIVFNVPKRRYWLFKGPIDSGKTTLAAGLLDLCGGKALNVNLPMERLTFELGVAIDQY
B12/A-62H (1a)-LTA    LHCIVFNVPKRRYWLFKGPIDSGKTTLAAGLLDLCGGKALNVNLPMERLTFELGVAIDQY
B9/RU13 (IV)_LTA      LHCIVFNVPKRRYWLFKGPIDSGKTTLAAGLLDLCGGKALNVNLPMERLTFELGVAIDQY
                      ************************************************************

BKV Dunlop (1a)-LTA   MVVFEDVKGTGAESKDLPSGHGINNLDSLRDYLDGSVKVNLEKKHLNKRTQIFPPGLVTM
B12/A-62H (1a)-LTA    MVVFEDVKGTGAESKDLPSGHGINNLDSLRDYLDGSVKVNLEKKHLNKRTQIFPPGLVTM
B9/RU13 (IV)_LTA      MVVFEDVKGTGAESKDLPSGHGINNLDSLRDYLDGSVKVNLEKKHLNKRTQIFPPGLVTM
                      ************************************************************

BKV Dunlop (1a)-LTA   NEYPVPKTLQARFVRQIDFRPKIYLRKSLQNSEFLLEKRILQSGMTLLLLLIWFRPVADF
B12/A-62H (1a)-LTA    NEYPVPKTLQARFVRQIDFRPKIYLRKSLQNSEFLLEKRILQSGMTLLLLLIWFRPVADF
B9/RU13 (IV)_LTA      NEYPVPKTLQARFVRQIDFRPKIYLRKSLQNSEFLLEKRILQSGMTLLLLLIWFRPVADF
                      ************************************************************

BKV Dunlop (1a)-LTA   ATDIQSRIVEWKERLDSEISMYTFSRMKYNICMGKCILDITREEDSETEDSGHGSSTESQ
B12/A-62H (1a)-LTA    ATDIQSRIVEWKERLDSEISMYTFSRMKYNICMGKCILDITREEDSETEDSGHGSSTESQ
B9/RU13 (IV)_LTA      AQDIQSRIVEWKERLDSEISMYTFSRMKYNICMGKCILDITREEDSETEDSGHGSSTESQ
                      * **********************************************************

BKV Dunlop (1a)-LTA   SQCSSQVSDTSAPAEDSQRSDPHSQELHLCKGFQCFKRPKTPPPK
B12/A-62H (1a)-LTA    SQCSSQVSDTSAPAEDSQRSDPHSQELHLCKGFQCFKRPKTPPPK
B9/RU13 (IV)_LTA      SQCSSQVSDTSAPAEDSQRLDPHSEELHLCKGFQCFKRPKTPPPK
                      ******************* ****:********************
```

<u>Note</u>

Sequence 1: Dunlop (1a) -LTA
Sequence 2: B9/RU13 (IV) -LTA
Sequence 3: B12/A-62H (1a)-LTA

<u>Alignment Score</u>

Sequences (1:2) Aligned. Score: 98.46%
Sequences (1:3) Aligned. Score: 99.65%
Sequences (2:3) Aligned. Score: 98.46%

Supplementary Figure S11. Multiple sequence alignment using ClustalW for large T-antigen protein. Overall protein similarity score was high (more than 98%) however, there were amino acid substitutions and are highlighted.

Large T antigen

BKV Dunlop (1a)

Rank	Cscore^{LB}	PDB Hit	TM-score	RMSD^{a}	IDEN^{a}	Cov.	BS-score	Lig. Name	Download Complex	Predicted binding site residues
1	0.51	1svmA	0.615	0.63	0.773	0.619	1.84	ATP	Download	289,320,321,322,323,324,325,326,366,421,440,441,442,445,446,449,456
2	0.38	2h1l5	0.615	0.79	0.774	0.621	1.84	PEPTIDE	Download	162,163,231,234,235,237,238,246,307,308,309,346,347,350,387,388,389,390,391,395,396,398,400,405,408,409,410,426,427,428,432
3	0.38	2h1l3	0.613	0.81	0.773	0.619	1.84	PEPTIDE	Download	176,178,179,181,182,183,202,204,220,221,222,223,225,226,320,325,338,339,341,343,344,345,347,348,351,352,365,366,368,375,376,377,378,404,405,421,423,459,462

B9/RU13 (IV)

Rank	Cscore^{LB}	PDB Hit	TM-score	RMSD^{a}	IDEN^{a}	Cov.	BS-score	Lig. Name	Download Complex	Predicted binding site residues
1	0.51	1svmA	0.611	0.97	0.776	0.619	1.87	ATP	Download	289,320,321,322,323,324,325,326,366,421,440,441,442,445,446,449,456
2	0.38	2h1l5	0.610	1.11	0.777	0.621	1.81	PEPTIDE	Download	162,163,231,234,235,237,238,246,307,308,309,346,347,350,387,388,389,390,391,395,396,398,400,405,408,409,410,426,427,428,432
3	0.38	2h1l3	0.608	1.13	0.776	0.619	1.81	PEPTIDE	Download	176,178,179,181,182,183,202,204,220,221,222,223,225,226,320,325,338,339,341,343,344,345,347,348,351,352,365,366,368,375,376,377,378,404,405,421,423,459,462

B12/A-62H (1a)

Rank	Cscore^{LB}	PDB Hit	TM-score	RMSD^{a}	IDEN^{a}	Cov.	BS-score	Lig. Name	Download Complex	Predicted binding site residues
1	0.66	1svmA	0.613	0.84	0.773	0.619	1.87	ATP	Download	289,320,321,322,323,324,325,326,366,421,440,441,442,445,446,449,456
2	0.38	2h1l5	0.613	0.98	0.774	0.621	1.83	PEPTIDE	Download	162,163,231,234,235,237,238,246,307,308,309,346,347,350,387,388,389,390,391,395,396,398,400,405,408,409,410,426,427,428,432
3	0.38	2h1l3	0.611	0.98	0.773	0.619	1.82	PEPTIDE	Download	176,178,179,181,182,183,202,204,220,221,222,223,225,226,320,325,338,339,341,343,344,345,347,348,351,352,365,366,368,375,376,377,378,404,405,421,423,459,462

Supplementary Figure S12. Predicted residue binding sites for Large T antigen protein in reference Dunlop (1a) strain B9/RU13 (IV) and B12/A-62H (1a) from two BKV+ patient's described in this study. Predicted binding sites for ligand ATP are highlighted with circle. The residue binding sites for Dunlop (1a) and other BKV sequences were predicted using the COFACTOR algorithm in I-TASSER Suite.

Supplementary Table S1. Number of virus reads in percent counts.

Group	Sample	Polyomaviridae	Anelloviridae	Herpesviridae	Papillomaviridae	Adenoviridae	Other viruses	Unclassified viruses
BKV+	B3	100.00	0.00	0.00	0.00	0.00	0.00	0.00
	B4	99.84	0.13	0.00	0.02	0.00	0.00	0.00
	B5	99.93	0.00	0.00	0.00	0.06	0.00	0.00
	B6	100.00	0.00	0.00	0.00	0.00	0.00	0.00
	B7	99.58	0.42	0.00	0.00	0.00	0.00	0.00
	B8	99.99	0.01	0.00	0.00	0.00	0.00	0.00
	B9	100.00	0.00	0.00	0.00	0.00	0.00	0.00
	B10	99.99	0.01	0.00	0.00	0.00	0.00	0.00
	B11	99.89	0.10	0.00	0.01	0.00	0.00	0.00
	B12	100.00	0.00	0.00	0.00	0.00	0.00	0.00
	B13	100.00	0.00	0.00	0.00	0.00	0.00	0.00
	B15	99.97	0.02	0.01	0.00	0.00	0.00	0.00
BKV-	B16	8.44	91.34	0.00	0.00	0.00	0.00	0.21
	B17	61.07	38.86	0.01	0.00	0.00	0.00	0.06
	B18	0.78	99.18	0.03	0.00	0.00	0.01	0.00
	B19	13.70	80.00	0.12	0.00	6.11	0.05	0.01
	B20	1.69	1.69	0.00	11.86	0.00	3.39	81.36
	B21	0.00	0.00	0.00	25.00	25.00	25.00	25.00
	B22	66.67	0.00	0.00	0.00	0.00	33.33	0.00
	B23	18.18	0.00	0.00	36.36	18.18	18.18	9.09
	B24	11.11	0.00	0.00	33.33	33.33	11.11	11.11
	B25	0.00	37.50	0.00	0.00	0.00	37.50	25.00
	B26	0.00	91.78	0.00	5.48	1.37	1.37	0.00
	B27	6.67	13.33	0.00	26.67	20.00	0.00	33.33
	B28	0.00	0.00	0.00	0.00	50.00	0.00	50.00
	B29	0.00	0.00	0.00	3.85	80.77	11.54	3.85
	B30	0.00	0.00	0.00	64.29	21.43	7.14	7.14

The relative abundance values are calculated form the total read counts for each virus.

Supplementary Table S2a. BKV virus count and subtype distribution in BKV+ kidney transplant group.

Sample	Reference	Subtype	Genome Size (bp)	Virus count[#]
B3 (BKV+)	BKV J2B-2	Ib1	5,141	192,450
B4 (BKV+)	BKV A-47H	Ib2	5,141	193,513
B5 (BKV+)	BKV SJH-LG-309	Ib1	5,134	28,544
	BKV J2B-2	Ib1	5,141	27,932
	BKV SJH-LG-304	Ib1	5,141	14,011
	BKV VP1M26	I	5,141*	12,628
	BKV WT	Ia	5,216	12,424
	BKV SJH-LG-306	Ib1	5,141	11,973
	BKV VP1M27	I	5,141*	10,583
	Human Papovavirus BK	-	5,153	3,836
	BKV ANY-63	Ic	5,142	2,612
	BKV SUSH/SGPGI	-	5,141*	2,064
	BKV HI-u8	Ib1	5,132	874
B6 (BKV+)	BKV SJH-LG-309	Ib1	5,134	79,134
	BKV SJH-LG-306	Ib1	5,141	46,619
	BKV J2B-2	Ib1	5,141	39,167
	BKV WT	Ia	5,216	15,444
	BKV Tun-36	-	5,141*	7,980
	BKV_ISR_E_V7.1	-	5,141*	815
	BKV VP1M26	I	5,141*	71
B7 (BKV+)	BKV LAB27	Ib1	5,141	190,748
B8 (BKV+)	BKV LAB27	Ib1	5,141	192,440
B9 (BKV+)	BKV RU13	IV	5,146	204,955
	BKV SJH-LG-309	Ib1	5,134	725
B10 (BKV+)	BKV A-47H	Ib2	5,141	112,576
B11 (BKV+)	BKV RU13	IV	5,146	1
	BKV A-47H	Ib2	5,141	494
B12 (BKV+)	BKV A-62H	Ia	5,141	51,398
B13 (BKV+)	BKV A-62H	Ia	5,141	100,447
	BKV WT	Ia	5,216	20,110
	BKV for LT	-	4,963	14,247
	BKV-D	Ia	5,172	8,229
	BKV VP1M23	I	5,141*	3,923
	BKV CAF-2	-	5,141*	3,130
	BKV VP1M27	I	5,141*	98
	BKV isolate patient 107	-	5,141*	17
	BKV SJH-LG-308	Ib2	5,141	4
B15 (BKV+)	BKV A-62H	Ia	5,141	4,232
	BKV WT	Ia	5,216	2,233

[#]Virus count: Reads per kb per million reads (RPKM) = (10^9 * C)/(N * L), where C = Number of reads mapped to BK virus, N = Total reads in the sample used for analysis, L = Genome length in base-pairs for BK virus (from NCBI Genome). *Predicted average genome size. [-] Subtype not known.

Supplementary Table S2b. BKV virus count and subtype distribution in BKV- kidney transplant group.

Sample	Reference	Subtype	Genome Size (bp)	Virus count#
B16 (BKV-)	BKV A-62H	Ia	5141	31
	BKV ANY-63	Ic	5142	11
	BKV BKV-D	Ia	5172	26
	BKV SJH-LG-168	Ib1	5141	27
	BKV SJH-LG-306	Ib1	5141	3
	BKV SJH-LG-309	Ib1	5134	15
B17 (BKV-)	BKV A-62H	Ia	5141	1453
	BKV SJH-LG-168	Ib1	5141	11
	BKV SJH-LG-309	Ib1	5134	176
	BKV J2B-9	Ib2	5141	50
B18 (BKV-)	BKV A-62H	Ia	5141	41
	BKV J2B-2	Ib1	5141	2
	BKV VNM-9	Ib1	5141	16
B19 (BKV-)	BKV A-62H	Ia	5141	3
	BKV J2B-2	Ib1	5141	110

#Virus count: Reads per kb per million reads (RPKM) = (10^9 * C)/(N * L), where C = Number of reads mapped to BK virus, N = Total reads in the sample used for analysis, L = Genome length in base-pairs for BK virus (from NCBI Genome).

Supplementary Table S3. JC virus count and subtype distribution in BKV+ and BKV- samples.

Sample	Reference	Subtype	Genome Size (bp)	Virus count#
B5 (BKV+)	JCV BJ-1	7B-1	5121*	0.5
	JCV ZJ-1	7B-1	5120	3.4
B6 (BKV+)	JCV BJ-1	7B-1	5121*	0.3
B13 (BKV+)	JCV OH-1	-	5147	1074
	JCV ML-2	-	5116	3.8
	JCV ZJ-1	7B-1	5120	1.2
	JCV BJ-1	7B-1	5121*	0.3
	JCV JAL	7A; SC-f	5121*	0.2
B15 (BKV+)	JCV ML-2	-	5116	2588
	JCV ZJ-1	7B-1	5120	4576
	JCV/5386/KW	-	5121*	2.8
	JCV SL-15	-	5119	1.6
B16 (BKV-)	JCV SP-1	1B	5122	8251
B17 (BKV-)	JCV SP-1	1B	5122	8246
	JCV CPN1	-	5121*	790
	JCV 173FLC-05	-	5121*	312
	JCV VSO-2	-	5121	304
	JCV 603	-	5121*	59
B18 (BKV-)	JCV OH-1	-	5147	0.3
	JCV 9	-	5121*	0.3
	JCV ZJ-1	7B-1	5120	0.3
	JCV176FLC-10	-	5121*	0.2
	JCV 406	-	5121*	0.2
B19 (BKV-)	JCV ZJ-1	7B-1	5120	2.2
	JCV RJ/2005-07	-	5121*	1.5
	JCV UK-1	1B	5122	1.4
	JCV 183FLC-01	-	5121*	1.0
	JCV MC-8	-	5121	0.9
	JCV 149URNFL-13	-	5121*	0.8
	JCV FL-8	1	5121	0.5

#Virus count: Reads per kb per million reads (RPKM) = (10^9 * C)/(N * L), where C = Number of reads mapped to JC virus, N = Total reads in the sample used for analysis, L = Genome length in base-pairs for JC virus (from NCBI Genome).
*Predicted average genome size. – Subtype not known.

Supplementary Table S4a. TT virus count and subtype distribution in BKV+ kidney transplant group.

Sample	Reference	Genome Size (bp)	Virus count#
B4 (BKV+)	TTV HD14h	3,725	275.1
	TTV sle2552	1,509	179.5
	TTVyon-LC011	3,675	15.6
	TTV genotype 23	3,792	13.5
	TTV 3CR11	3,725*	10.5
	TTV SIA109	3,260	5.3
B5 (BKV+)	TTV Polish isolate P/1C1	3,756	11.3
	TTV isolate L02	3,206	0.4
B6 (BKV+)	TTV ViPi04	3,774	1.9
	TTV HD22 (rheu112)	3,816	0.9
	TTV HD11 sle2037	3,814	0.6
	TTV CT25F	3,726	0.3
B7 (BKV+)	TTV 21 TCHN-B	3,153	1464.4
	TTV 5 TCHN-C1	3,229	416.0
	TTV CT25F	3,726	340.3
	TTV L018	3,725*	210.8
	TTV genotype 23	3,792	94.9
	TTV 20 SAa-10	3,234	87.8
	TTV 29 TTVyon-KC009	3,676	46.4
	TTV JA10	3,539	41.3
	TTV TYM9-cDNA d-1	2,297	40.6
	TTV JT19F	3,676	25.2
	TTV 3 HEL32	3,748	20.7
	TTV 19 SANBAN	3,808	11.0
	TTV 3CR11	3,725*	7.6
	TTV mini LIL-y1	2,887	3.4
	TTV mini 1 TLMV-CBD279	2,856	2.8
	TTV mini TLMV-CLC205	2,841	2.8
B8 (BKV+)	TTV SAf-09	3,155	16.0
	TTV L018	3,725*	12.6
	TTV 5 TCHN-C1	3,229	6.2
	TTV 3 HEL32	3,748	5.6
	TTV mini LIL-y1	2,887	1.1
B10 (BKV+)	TTV midi Pt-TTMDV225-2	3,269	581.2
	TTV midi MDJN91	3,183	19.6
B13 (BKV+)	TTV midi Pt-TTMDV225-2	3,269	62.5
	TTV TUPB	3,817	12.3
	TTV midi MDJN91	3,183	2.0
	TTV ViPi04	3,774	1.8
	TTV CT25F	3,726	1.2
B15 (BKV+)	TTV TUPB	3,817	32.4
	TTV KC005/2-21E	3,725*	0.4

#Virus count: Reads per kb per million reads (RPKM) = (10^9 * C)/(N * L), where C = Number of reads mapped to TT virus, N = Total reads in the sample used for analysis, L = Genome length in base-pairs for TTV (from NCBI Genome). *Predicted average genome size.

Supplementary Table S4b. TT virus count and subtype distribution in BKV- kidney transplant group.

Sample	Reference	Genome Size (bp)	Virus count#
B16 (BKV-)	TTV HD14h	3727	6795.2
	TTV tth4	3772	6476.5
	TTV tth26	3756	2628.5
	TTV tth21	3741	2516.0
	TTV-HD20b	3834	2496.4
	TTV-HD17	3756	2436.0
	TTV ZC-1998-2	3725	796.7
	TTV midi MDJHem3-2	3197	705.3
	TTV midi MDJN1	3223	298.3
	TTV midi 1 MD1-073	3242	284.8
	TTV-HD20d	3880	254.9
	TTV midi Pt-TTMDV210	3257	254.3
	TTV midi L67NA1	3725	250.0
	TTV 27 CT23F	3729	123.6
	TTV midi MDJN97	3223	121.9
	TTV-HD20f	3881	109.7
	TTV midi L83NA1	3725	84.6
	TTV JT19F	3676	45.2
	TTV midi MDJN51	3187	20.8
	TTV ViPi08	3479	7.5
	TTV CT30F	3570	3.9
	TTV CT25F	3726	3.2
	TTV mini 5 TGP96	2908	3.2
	TTV CT43-14	3725	1.8
	TTV JT41F	3727	1.6
	TTV midi L92NA1	3725	0.6
B17 (BKV-)	TTV CT25F	3726	4167.6
	TTV 3 HEL32	3748	1343.0
	TTV midi MDJHem5	3205	1436.9
	TTV tth29	3700	1101.0
	TTV mini 5 TGP96	2908	867.6
	TTV 16 TUS01	3818	493.7
	TTV midi MDJN69	3230	110.0
	TTV tth21	3741	8.8
	TTV HD20a	3878	5.5
	TTV HD20f	3881	1.2
	TTV HD14i	3726	1.1
	TTV CT25-05	3725	1.0
	TTV HD14d	3728	0.8
	TTV-HD14h	3727	0.8

B18 (BKV-)	TTV HD22	3816	402.6
	TTV ViPi08	3479	343.0
	TTV JT41F	3727	69.2
	TTV sle2550	3725	21.9
	TTV KC009/2-3G	3725	13.0
	TTV ViPi04	3774	6.6
	TTV sle2552	1509	1.7
	TTV SIA109	3260	0.3
	TTV HD24f	3758	0.3
	TTV HD13c gsB21.51	3749	0.1
B19 (BKV-)	TTV SIA109	3260	582.2
	TTV 24 SAa-01	3246	226.0
	TTV CT25F	3726	157.3
	TTV HD15d	3700	145.2
	TTV Polish isolate P/1C1	3756	21.6
	TTV KC009/2-3G	3725	8.3
	TTV HD13c gsB21.51	3749	2.8
	TTV L02	3206	1.5
	TTV 3CR08	3725	1.5
	TTV HD13a gsB20.33	3749	1.2
	TTV T3PB	3838	0.9
	TTV HD20f	3881	0.2

[#]Virus count: Reads per kb per million reads (RPKM) = (10^9 * C)/(N * L), where C = Number of reads mapped to TT virus, N = Total reads in the sample used for analysis, L = Genome length in base-pairs for TTV. * Predicted average genome size.

Supplementary Table S5. Number of virus subtypes in BKV+ and BKV- groups.

**Sample name**	**BKV**	**JCV**	**TTV**	***Sample average***
B3	1.0	0.0	0.0	*0.3*
B4	1.0	0.0	6.0	*2.3*
B5	11.0	2.0	2.0	*5.0*
B6	7.0	1.0	4.0	*4.0*
B7	1.0	0.0	17.0	*6.0*
B8	1.0	0.0	5.0	*2.0*
B9	2.0	0.0	0.0	*0.7*
B10	1.0	0.0	2.0	*1.0*
B11	2.0	0.0	0.0	*0.7*
B12	1.0	0.0	0.0	*0.3*
B13	9.0	5.0	5.0	*6.3*
B15	2.0	4.0	2.0	*2.7*
Group Average	*3.3*	*1.0*	*3.6*	2.6
B16	6.0	1.0	26.0	*11.0*
B17	4.0	5.0	14.0	*7.7*
B18	3.0	5.0	10.0	*6.0*
B19	2.0	7.0	11.0	*6.7*
B20	1.0	0.0	1.0	*0.7*
B21	0.0	0.0	0.0	*0.0*
B22	0.0	1.0	0.0	*0.3*
B23	1.0	0.0	0.0	*0.3*
B24	1.0	0.0	0.0	*0.3*
B25	0.0	0.0	1.0	*0.3*
B26	0.0	0.0	1.0	*0.3*
B27	1.0	0.0	1.0	*0.7*
B28	0.0	0.0	0.0	*0.0*
B29	0.0	0.0	0.0	*0.0*
B30	0.0	0.0	0.0	*0.0*
Group Average	*1.3*	*1.3*	*4.3*	2.3

Supplementary Table S6. RM plot statistics of VP1 protein for reference DUNLOP and two BKV+ samples from BKV+ kidney transplant group.

RM Plot Statistics	DUNLOP	B9	B12
	**Residues**		
Residues in most favored regions [A,B,L]	211 (70.3%)	204 (68.0%)	211 (70.3%)
Residues in additional allowed regions [a,b,l,p]	80 (26.7%)	82 (27.3%)	76 (25.3%)
Residues in generously allowed regions [~a,~b,~l,~p]	8 (2.7%)	11 (3.7%)	9 (3.0%)
Residues in disallowed regions	1 (0.3%)	3 (1.0%)	4 (1.3%)
Number of non-glycine and non-proline residues	300 (100%)	300 (100%)	300 (100%)
Number of end-residues (excl. Gly and Pro)	2	2	2
Number of glycine residues (shown as triangles)	31	31	31
Number of proline residues	28	29	28
Total number of residues	361	362	361

Supplementary Table S7. Nucleotide substitution observed in VP2 protein in sample B10 (BKV+ sample) compared to reference BKV genome.

Reference strain/ Sample	Subtype /Subgroup	Dunlop position	Amino acid	Nucleotide substitutions	Translated protein code
DUNLOP	Ia	1257	423	**<u>T</u>**AT	Y (Tyr)
A-47H	Ib2			TAT	Y (Tyr)
B10	Ib2			**<u>C</u>**AT	H (His)
DUNLOP	Ia	2670	895	ACA	T (Thr)
A-47H	Ib2			T**<u>G</u>**A	*
B10	Ib2			T**<u>A</u>**A	*

*Stop codon. Nucleotide substitutions are indicated in bold and underline.

Supplementary Table S8. RM plot statistics of VP2 protein for reference DUNLOP and two BKV+ samples from BKV+ kidney transplant group.

RM Plot Statistics	DUNLOP	B9	B12
	Residues		
Residues in most favored regions [A,B,L]	206 (66.7%)	207 (67.0%)	221 (71.5%)
Residues in additional allowed regions [a,b,l,p]	77 (24.9%)	77 (24.9%)	74 (23.9%)
Residues in generously allowed regions [~a,~b,~l,~p]	16 (5.2%)	16 (5.2%)	8 (2.6%)
Residues in disallowed regions	10 (3.2%)	9 (2.9%)	6 (1.9%)
Number of non-glycine and non-proline residues	309 (100%)	309 (100%)	309 (100%)
Number of end-residues (excl. Gly and Pro)	2	2	2
Number of glycine residues (shown as triangles)	25	25	25
Number of proline residues	15	15	15
Total number of residues	351	585	585

Supplementary Table S9. RM plot statistics of Large T antigen protein for reference DUNLOP and two BKV+ samples from BKV+ kidney transplant group.

**RM Plot Statistics**	**DUNLOP**	**B9**	**B12**
	**Residues**		
Residues in most favored regions [A,B,L]	393 (73.9%)	381 (71.6%)	397 (74.6%)
Residues in additional allowed regions [a,b,l,p]	104 (19.5%)	105 (19.7%)	102 (19.2%)
Residues in generously allowed regions [~a,~b,~l,~p]	20 (3.8%)	28 (5.3%)	21 (3.9%)
Residues in disallowed regions	15 (2.8%)	18 (3.4%)	12 (2.3%)
Number of non-glycine and non-proline residues	532 (100%)	532 (100%)	532 (100%)
Number of end-residues (excl. Gly and Pro)	2	2	2
Number of glycine residues (shown as triangles)	25	25	25
Number of proline residues	26	26	26
Total number of residues	585	585	585

Supplementary Table S10. Virus identified in BKV+ and BKV- group.

Virus	BKV+												BKV -														
	**B3**	**B4**	**B5**	**B6**	**B7**	**B8**	**B9**	**B10**	**B11**	**B12**	**B13**	**B15**	**B16**	**B17**	**B18**	**B19**	**B20**	**B21**	**B22**	**B23**	**B24**	**B25**	**B26**	**B27**	**B28**	**B29**	**B30**
Mardivirus	0.0	0.0	0.0	0.0	0.0	0.0	0.0	0.0	0.0	0.0	0.0	0.0	0.0	0.0	0.0	0.0	0.0	0.0	0.0	0.0	0.0	0.0	0.0	0.0	0.0	0.0	0.0
Cytomegalovirus	0.0	0.0	0.0	0.0	0.0	0.0	0.0	0.0	0.0	0.0	0.0	0.0	0.0	0.0	0.0	0.1	0.0	0.0	0.0	0.0	0.0	0.0	0.0	0.0	0.0	0.0	0.0
Alpha-Papillomavirus n	0.0	0.0	0.0	0.0	0.0	0.0	0.0	0.0	0.0	0.0	0.0	0.0	0.0	0.0	0.0	0.0	0.0	0.0	0.0	0.0	0.0	0.0	0.0	0.0	0.0	0.0	6.7
Beta-Papillomavirus n	0.0	0.0	0.0	0.0	0.0	0.0	0.0	0.0	0.0	0.0	0.0	0.0	0.0	0.0	0.0	0.0	0.1	0.0	0.0	13.3	28.6	0.0	2.6	16.7	0.0	0.0	26.7
Gamma-Papillomavirus n	0.0	0.0	0.0	0.0	0.0	0.0	0.0	0.0	0.0	0.0	0.0	0.0	0.0	0.0	0.0	0.0	0.1	0.0	0.0	0.0	0.0	0.0	1.3	0.0	0.0	0.0	0.0
Chlorovirus	0.0	0.0	0.0	0.0	0.0	0.0	0.0	0.0	0.0	0.0	0.0	0.0	0.0	0.0	0.0	0.0	0.0	0.0	0.0	0.0	0.0	0.0	0.0	0.0	0.0	0.0	0.0
Polyomavirus BK	100.0	99.8	99.9	100.0	99.6	100.0	100.0	100.0	99.9	100.0	99.9	99.9	0.0	14.7	67.1	11.2	96.7	20.0	0.0	26.7	28.6	8.3	0.0	0.0	4.3	0.0	0.0
Polyomavirus JC	0.0	0.0	0.0	0.0	0.0	0.0	0.0	0.0	0.0	0.0	0.1	0.1	8.2	51.6	0.0	1.1	0.0	0.0	40.0	0.0	0.0	0.0	0.0	0.0	0.0	0.0	0.0
Totivirus	0.0	0.0	0.0	0.0	0.0	0.0	0.0	0.0	0.0	0.0	0.0	0.0	0.0	0.0	0.0	0.0	0.1	0.0	0.0	0.0	0.0	8.3	0.0	0.0	0.0	0.0	0.0
Lentivirus	0.0	0.0	0.0	0.0	0.0	0.0	0.0	0.0	0.0	0.0	0.0	0.0	0.0	0.0	0.0	0.0	0.0	0.0	0.0	0.0	0.0	8.3	0.0	0.0	0.0	0.0	0.0
Alpha-torquevirus	0.0	0.0	0.0	0.0	0.2	0.0	0.0	0.0	0.0	0.0	0.0	0.0	3.7	2.7	1.6	3.6	0.0	0.0	0.0	0.0	0.0	0.0	6.6	0.0	0.0	0.0	0.0
Beta-torquevirus	0.0	0.0	0.0	0.0	0.0	0.0	0.0	0.0	0.0	0.0	0.0	0.0	0.0	0.1	0.0	0.0	0.0	0.0	0.0	0.0	0.0	0.0	0.0	0.0	0.0	0.0	0.0
Gamma-torquevirus	0.0	0.0	0.0	0.0	0.0	0.0	0.0	0.0	0.0	0.0	0.0	0.0	0.0	0.0	0.0	0.0	0.0	0.0	0.0	0.0	0.0	0.0	0.0	0.0	0.0	0.0	0.0
Begomovirus	0.0	0.0	0.0	0.0	0.0	0.0	0.0	0.0	0.0	0.0	0.0	0.0	0.0	0.0	0.0	0.0	0.0	0.0	0.0	0.0	0.0	0.0	0.0	0.0	0.0	0.0	0.0
Ilarvirus	0.0	0.0	0.0	0.0	0.0	0.0	0.0	0.0	0.0	0.0	0.0	0.0	0.0	0.0	0.0	0.0	0.0	0.0	0.0	0.0	0.0	0.0	0.0	0.0	0.0	0.0	0.0
Sapovirus	0.0	0.0	0.0	0.0	0.0	0.0	0.0	0.0	0.0	0.0	0.0	0.0	0.0	0.0	0.0	0.0	0.0	20.0	0.0	6.7	0.0	0.0	1.3	0.0	0.0	10.0	0.0
Enterovirus	0.0	0.0	0.0	0.0	0.0	0.0	0.0	0.0	0.0	0.0	0.0	0.0	0.0	0.0	0.0	0.0	0.0	0.0	0.0	0.0	0.0	0.0	0.0	0.0	0.0	0.0	0.0
Mastadenovirus	0.0	0.0	0.1	0.0	0.0	0.0	0.0	0.0	0.0	0.0	0.0	0.0	0.0	0.0	0.2	0.0	0.1	0.0	20.0	20.0	0.0	8.3	3.9	8.3	91.3	30.0	20.0
Other viruses	0.0	0.0	0.0	0.0	0.0	0.0	0.0	0.0	0.0	0.0	0.0	0.0	0.0	0.0	0.0	0.1	0.1	20.0	20.0	13.3	14.3	25.0	1.3	0.0	0.0	30.0	6.7
Unclassified Papillomaviridae	0.0	0.0	0.0	0.0	0.0	0.0	0.0	0.0	0.0	0.0	0.0	0.0	0.0	0.0	0.0	0.0	0.3	20.0	0.0	13.3	14.3	0.0	1.3	16.7	0.0	10.0	26.7
Unclassified Anelloviridae	0.0	0.1	0.0	0.0	0.2	0.0	0.0	0.0	0.1	0.0	0.0	0.0	87.9	30.8	31.1	83.8	0.1	0.0	0.0	0.0	0.0	25.0	81.6	16.7	0.0	0.0	0.0
Unclassified viruses	0.0	0.0	0.0	0.0	0.0	0.0	0.0	0.0	0.0	0.0	0.0	0.0	0.2	0.1	0.0	0.0	2.6	20.0	0.0	6.7	14.3	16.7	0.0	41.7	4.3	10.0	6.7

The relative abundance values are calculated form the total read counts for each virus.
